# Virulence Evolution in Host Populations with Heterogeneous Immune Responses: The Weakening of the Uncommon Weak

**DOI:** 10.1007/s11538-026-01673-9

**Published:** 2026-06-09

**Authors:** Carles Barril, Marina Vegué

**Affiliations:** 1 Departament de matemàtiques, Universitat Autònoma de Barcelona, Barcelona, Spain; 2 Departament de matemàtiques, Universitat Politècnica de Catalunya, Barcelona, Spain

**Keywords:** Virulence evolution, Heterogeneous immunity, Host-Parasite dynamics, Lyapunov functions, 92D15, 92D30, 34D23

## Abstract

How and to what extent different factors in pathogen-host dynamics shape virulence has been largely debated due to evolutionary forces acting in opposite directions. Here we present a model of pathogen-host dynamics with the aim of studying how competition between pathogens and the immune response of the host affect virulence. The model describes, and links, the pathogen dynamics inside individual hosts and the dynamics of the susceptible and infected individuals in the host population. We first show that when the hosts have an adequate immune response (so that they completely recover from the infection on a short time scale), pathogens tend to increase their virulence. On the contrary, if the hosts are vulnerable (meaning that they will eventually die from the infection), low virulences will be selected. We then move to a richer scenario in which the host population is mixed and contains a fraction *p* of vulnerable individuals. We see that the selected virulence exhibits biologically relevant behaviors as *p* takes intermediate values.

## Introduction

The evolution of virulence, understood as the damage caused by a pathogen to its host, has been object of intense debate in the last century (Bull 1994; Read 1994). The issue is that virulence is *per se* disadvantageous for the parasite: a higher virulence implies a decline in the host population, which is the only resource that the pathogen uses to survive. In other words, killing the host too fast or impairing too much its reproduction can prevent the parasite from successively replicating and transmitting. Based on this idea, the first discussions on the problem of virulence evolution concluded that pathogens would unavoidably become less and less virulent in time. This is known as the “avirulence hypothesis" (Alizon et al. 2009). Despite several observations provided empirical evidence that some new host-parasite relations are indeed virulent, a universal trend towards avirulence could not be confirmed experimentally (Alizon et al. 2009; Ball 1943). From the 1940s, important criticisms were raised by some authors (Ball 1943), and a few decades later a new view was imposed over the old avirulence hypothesis, especially after the work of Ewald (1983) and Anderson and May (1982), who discussed situations in which virulence could have an adaptive role and hence persist (see Méthot (2012) for an insightful historical perspective to the avirulent hypothesis).

The new view stated that, although virulence is disadvantageous *per se*, it tends to be correlated with other traits that are themselves advantageous for the pathogen. This is so because virulence is directly linked to the pathogen’s growth process within the host, and, as such, it cannot be disentangled from other traits also related to it. For example, virulence tends to correlate with replication rate: a strain that replicates faster inside a host, also tends to produce a greater harm, simply because the pathogen’s replication is directly linked to cellular damage. Thus, increasing virulence induces a higher host damage but it may concomitantly imply a higher replication rate and indirectly result in a greater ability to grow and be transmitted. This led to the idea that there are different traits which are beneficial for the pathogen and that necessarily correlate with virulence, which makes it not possible for evolution to reduce virulence without reducing them too. In short: pathogens have to deal with a sort of “trade-off" between virulence and other traits, so the fittest strains might not necessarily be the ones that are less virulent. Alizon et al. (2009) refer to a collection of experimental studies that seem to support the presence of trade-offs in real host-parasite systems, even if these relationships are difficult to measure experimentally (Alizon and Michalakis 2015).

In this line, many authors share the view that the interdependency between virulence and the cellular and physiological mechanisms underlying the infection process makes it difficult to anticipate how virulence will evolve. Moreover, the particularities of each pathogen-host system might be determinant in shaping this evolution, with different systems evolving towards different degrees of virulence (Anderson and May 1982; Gandon and Day 2003; Alizon et al. 2009; Geoghegan and Holmes 2018). In order to shed some light on this issue, for every particular system one could attempt to plot its “trade-off curve", which describes how transmission rate and virulence relate to each other (as the parasite’s phenotype is varied). As Alizon et al. point out, the shape of this curve depends on the particularities of the infectious process considered, and it determines whether or not an optimal virulence can exist (i.e., a virulence level that maximizes pathogen’s fitness) (Alizon et al. 2009).

Trade-off relationships for a particular system can be found by formulating theoretical models that are able to capture the infectious dynamics at different levels, namely the within-host dynamics and and the population dynamics. Whereas the within-host dynamics describes how pathogens grow and compete within a host, the epidemic model describes how the infection shapes the number of infected and non-infected hosts as time goes on. Both levels are crucial for understanding the long-term evolution of parasites, and, by linking and/or nesting them, some of the relevant parameters at the population level (such as transmission rates, recovery rates and virulence) can be established in terms of pathogen properties (such as replication rates and degradation rates). The trade-offs at the population level are thus modeled using the pathogen dynamics in the hosts (Sasaki and Iwasa 1991; Gilchrist and Sasaki 2002; Ganusov et al. 2002; Mideo et al. 2008; André and Gandon 2006; Pugliese 2011; Martcheva et al. 2017). This strategy was already adopted by Sasaki and Iwasa in the nineties to study the optimal growth schedule of a pathogen throughout the infection. They showed that, to maximize transmissibility, the pathogen had to adjust its growth rate in a way that depends on the host age: virulence needs to be low when the host is young (so as not to kill it too early), but the pathogen must become more virulent as the host ages or weakens, since then the host is no longer useful as a reservoir. Within-host models are not only useful to explain the epidemic using information on the pathogen at the cellular level. As discussed in Mideo et al. (2008), these models may be essential when the infection dynamics in the host is affected by the state of the epidemic itself. This is, however, something we do not consider in the present work.

In this paper, we study the virulence evolution for a pathogen infecting a population of hosts structured according to the immunity level of its individuals. This gives us some clues on how the immune response of the hosts shapes pathogen virulence in the long term. We assume that a fraction of hosts can recover from the infection (we say that these hosts are *resistant*) and the rest cannot (we call them *vulnerable*). We can think that this reflects a scenario in which a vaccination campaign reaches only a fraction of the population, or a situation in which resource scarcity makes some individuals become vulnerable to some infections. The type of disease we have in mind is a chronic infection caused by a virus or a bacteria (Byndloss and Tsolis 2016). Although competition between pathogens inside each host is not taken into account in many within-host disease models (because it is assumed that the infection does not last enough for competition to play a role), in chronic infections like the ones experienced by vulnerable hosts, competition could be relevant. We take into account the effect of competition by incorporating it in the within-host dynamics and by linking the within-host and epidemic dynamics.

As pointed out in Antia and Lipsitch (1997) and Pfennig (1468), in immunocompetent hosts pathogens must replicate fast in order to optimize propagation prior to clearance, whereas in immunodepressed hosts fast replication can entail low host longevity and, consequently, inefficient propagation. Different pathogen traits are thus selected depending on the host particularities, and this makes the virulence to transmissibility trade-off be non-trivial in heterogeneous host populations. The main goal of this work is to obtain the trade-off associated to the dimorphic population described above and to discuss its biological implications. In particular, we show that low virulence levels are selected if vulnerable hosts are not too rare, whereas more virulent pathogens are selected if only a small fraction of the population is vulnerable. This implies that vulnerable hosts die more often due to the infection in the later case than in the former, an ecoepidemiological phenomenon (Venturino 2016) we call ‘the weakening of the uncommon weak’. The reason why resistant hosts favor more virulent pathogens (compared to vulnerable hosts) is similar to that given by Sasaki and Iwasa to explain why a pathogen must combine a low virulence phase with a high virulence phase to maximize transmission in aging hosts (Sasaki and Iwasa 1991). Resistant hosts can be identified with aged hosts, since they are not useful as reservoirs for the pathogen (because immunity kills the pathogen in resistant hosts and because the host is about to die in aged hosts), whereas vulnerable hosts play the role of young hosts.

The paper is organized as follows. In Section 2 we formulate an epidemiological model in which infected individuals are grouped into resistant and vulnerable. The fittest pathogen phenotype is then determined as a function of the model parameters. In Section 3, a within-host model is presented to describe how the pathogen population grows inside the hosts. The pathogen phenotype subject to natural selection is one of the parameters of this model, and it is related to the pathogen replication rate. We analyze which would be the selected phenotype if selection took place only within the host. In Section 4 we link the epidemiological model to the within-host model, which allows us to express the parameters of the former in terms of the dynamics of the later. By doing so we obtain trade-offs relating transmissibility and mortality that are based on the pathogen dynamics in the hosts. Finally, in Section 5 we show what level of virulence is selected as a function of the fraction of vulnerable hosts.

## Dynamics Within The Population of Hosts

We focus on epidemics in which infected individuals are immunized for the rest of their life after recovering from the infection. The model includes one compartment for the susceptible population and two compartments for the infected individuals. We distinguish two types of infected individuals: immunocompetent (resistant) and immunocompromised (vulnerable). The former are individuals which recover a few days after being infected. The later are individuals for which the infection becomes chronic and eventually kills them. The fraction of vulnerable hosts, *p*, is assumed to be a constant of the system and is a reflect of how effective the immune response is in the host population. We make this distinction between resistant and vulnerable hosts in order to study how the parameter *p* shapes the pathogen virulence that will be selected by evolution.

The dynamic equations governing the epidemics are the following:

1 $$\begin{aligned} \begin{aligned} S'(t) =&\; b - m S (t) - \left( k_1 I_1 (t)+k_2 I_2 (t)\right) S (t) \\ I_1'(t) =&\; (1-p)\left( k_1 I_1 (t)+k_2 I_2 (t)\right) S (t) - m I_1(t)-r I_1(t)\\ I_2'(t) =&\; p\left( k_1 I_1 (t)+k_2 I_2 (t)\right) S (t) -m I_2(t) - m_d I_2(t) \end{aligned} , \end{aligned}$$

where the meaning of the variables and parameters of the model can be found in Table 1. Notice that, from a mathematical point of view, the two hosts can be considered to be characterized by their transmission rates ($k_1$ and $k_2$) and their removal rates ($m_1:=m+r$ and $m_2:=m+m_d$). However, since we want to study how the induced mortality of vulnerable hosts evolves, we choose to work with parameters *m*, *r* and $m_d$ instead of $m_1$ and $m_2$.

The scenario studied here is similar to that analyzed in André and Gandon (2006), in which there is also a distinction between vulnerable and susceptible individuals. However, our model is simpler (the susceptible compartment is one-dimensional and infected individuals are not structured by infection age) and this allows us to establish global results on the pathogen’s evolutionary dynamics.

**Table 1:** Variables and parameters of system (1).

Variables	Description
*S*	number of susceptible hosts
$I_1$	number of infected hosts that are resistant
$I_2$	number of infected hosts that are vulnerable
Parameters	Description (units)
*b*	birth rate (hosts/day)
*m*	natural mortality rate (1/day)
$k_1$	infection rate from resistant hosts (1/(hosts · day))
$k_2$	infection rate from vulnerable hosts (1/(hosts · day))
*p*	fraction of vulnerable hosts
*r*	recovery rate for resistant hosts (1/day)
$m_d$	mortality rate due to the disease for vulnerable hosts (1/day)

In addition to the disease-free equilibrium (DFE) $\bar{P}_0 = (b/m,0,0)$, an endemic equilibrium (EE) $\bar{P} = (\bar{S},\bar{I}_1,\bar{I}_2)$ exists if, and only if, the *transmission potential*

$$ K:=(1-p)\frac{k_1}{m+r}+p\frac{k_2}{m+m_d} $$

satisfies bK>m. This endemic equilibrium is given by

2 $$\begin{aligned} \bar{S} = 1/K, \qquad \bar{I}_1 = \frac{1-p}{m+r} \left( b - \frac{m}{K} \right) , \qquad \bar{I}_2 = \frac{p}{m+m_d} \left( b - \frac{m}{K} \right) . \end{aligned}$$

When it exists (i.e., bK>m), the EE is a global attractor of the dynamics within the positive cone. Otherwise (i.e., bK≤m), the DFE is a global attractor of the dynamics (see Propositions 1 and 2 in the Appendix for a detailed proof of these results). Notice that a transmission potential equal to *K* means that an infected individual produces on average *SK* secondary infections provided that the susceptible population is fixed to *S*. In particular, $R_0:= b K/m$ is the so-called *reproduction number*: the expected number of secondary infections produced by a primary infected individual at the beginning of the infection (Diekmann et al. 2010; Van den Driessche 2017). Thus, the condition for the existence of the EE is equivalent to $R_0 > 1$.

Now we introduce a phenotypic variable *x* associated to the pathogen. The phenotype is assumed to affect both the infection rates ($k_1$ and $k_2$) and the induced mortality rate ($m_d$), but not the fraction of vulnerable hosts (*p*) nor the recovery rate of resistant hosts (*r*). Thus, the transmission potential now depends on *x* as

3 $$\begin{aligned} K(x):=(1-p)\frac{k_1(x)}{m+r}+p\frac{k_2(x)}{m+m_d(x)}, \end{aligned}$$

and the endemic equilibrium also depends on *x* consequently.

We first study which is the fittest phenotype *x*, i.e., the one favored by natural selection. To make this clearer, let us imagine that we have a resident pathogen whose phenotype is *x* and we wonder whether a mutant pathogen with phenotype *y* is able to proliferate and replace the resident one. At this point we need to describe what is the dynamics when we consider the two strains together, i.e. system (1) has to be extended so that it includes both pathogen populations. Such an extension is done by neglecting co-infection events, that is, assuming that every individual can be infected only once. Although this is a major simplification (there is no natural mechanism preventing that an individual infected with one strain could be infected a second time with another strain), we assume it for mathematical convenience. The two-strain system reads:

4 $$\begin{aligned} \begin{aligned} S'(t) =&\; b - m S (t) - \left( k_1(x) I_1 (t;x)+k_2(x) I_2 (t;x)\right. \\&\left. \qquad +k_1(y) I_1 (t;y)+k_2(y) I_2 (t;y)\right) S (t) \\ I_1'(t;x)&= (1-p)\left( k_1(x) I_1 (t;x)+k_2(x) I_2 (t;x)\right) S (t) - m I_1(t;x)-r I_1(t;x)\\ I_2'(t;x)&= p\left( k_1(x) I_1 (t;x)+k_2(x) I_2 (t;x)\right) S (t) - m I_2(t;x) - m_d(x) I_2(t;x) \\ I_1'(t;y)&= (1-p)\left( k_1(y) I_1 (t;y)+k_2(y) I_2 (t;y)\right) S (t) - m I_1(t;y)-r I_1(t;y)\\ I_2'(t;y)&= p\left( k_1(y) I_1 (t;y)+k_2(y) I_2 (t;y)\right) S (t) -m I_2(t;y) - m_d(y) I_2(t;y) \end{aligned} , \end{aligned}$$

where $I_1(t;z), I_2(t;z)$ are the number of resistant and vulnerable hosts that are infected by strain *z* at time *t*.

In the Appendix (Proposition 3) we show that if K(x)>K(y), the mutant *y* extinguishes. In fact, we show that the result is also true for a system in which *n* different phenotypes are present: only the population of phenotype *z* with the greatest *K*(*z*) can avoid extinction. That is, the principle of competitive exclusion applies (McGehee and Armstrong 1977; Bremermann and Thieme 1989), with susceptibles being the one-dimensional resource the strains compete for (see Metz et al. (1996) and Remark 4 in the Appendix). In agreement with what happens when there is a single strain, this phenotype will persist in the long term if, and only if, bK(z)>m. In this case, the number of susceptible, resistant infected and vulnerable infected hosts is given, as a function of the phenotype *z*, by

5 $$\begin{aligned} \bar{S}(z) = 1/K(z), \; \bar{I}_1(z) = \frac{1-p}{m+r} \left( b - \frac{m}{K(z)} \right) , \; \bar{I}_2(z) = \frac{p}{m+m_d(z)} \left( b - \frac{m}{K(z)} \right) . \end{aligned}$$

It follows that the phenotype *z* maximizing *K*(*z*) cannot be invaded by any mutant, so the fittest phenotype is the one that maximizes the transmission potential *K*. Thus, if new mutations appear as time goes on, we expect natural selection to drive pathogen evolution towards the phenotype *z* such that *K*(*z*) is maximal (or locally maximal if mutations are small). Such an extremization principle exists because the hosts are assumed to behave independently (i.e., there is no competition, mutualism, or any other ecological relation between them) and the rate at which a host “contacts” other hosts is assumed to increase linearly with the total population (this leaves out, for instance, any scenario in which hosts tend to avoid contacts when many individuals are infected). If these assumptions were relaxed (as in Svennungsen and Kisdi (2009)), the system could exhibit evolutionary branching and evolutionarily stable dimorphisms.

As shown in Eq. (3), the transmission potential *K*(*x*) depends on the phenotype *x* through functions $k_1(x)$, $k_2(x)$ and $m_d(x)$. Here we define the virulence associated to a strain *x* (which should be a measure of the harm produced by the virus to its hosts) as the induced mortality rate $m_d(x)$. Thus, all the previous results confirm the intuitive idea that for the pathogen it is advantageous to exhibit large transmission rates $k_1(x), k_2(x)$ while keeping the virulence $m_d(x)$ low.

To study what is the selected virulence (i.e., the value of $m_d(x)$ such that *x* is the phenotype maximizing *K*(*x*)), we need to know how $k_1(x), k_2(x)$ and $m_d(x)$ depend on *x*. This information will also shed light on the mutual dependence between parameters $k_1(x), k_2(x)$ and $m_d(x)$ (that is, on transmission-virulence trade-offs). To do so, we consider that, for a given strain *x*, $k_1(x), k_2(x)$ and $m_d(x)$ are shaped by the pathogen dynamics within an infected host. This is a reasonable assumption, for transmission rates and virulence depend on how pathogens grow within its host.

In the next section we propose a model for the pathogen dynamics within a host and we analyze its asymptotic behavior. Later on, we use these results to link the within-host dynamics with the phenotype-specific parameters $k_1(x), k_2(x)$ and $m_d(x)$.

## Dynamics Within The Host

We assume that the population of pathogens with phenotype *x* in a host, denoted by *V*, is determined by

6 $$\begin{aligned} \dot{V} = \tilde{\beta }(V,x)V - \tilde{\mu }(V,x)V, \end{aligned}$$

where functions $\tilde{\beta }(V,x)$ and $\tilde{\mu }(V,x)$ are the replication and elimination rates of the pathogens, respectively, and may depend on *V* and on the pathogen’s phenotype *x*. See Smith and Holt (1996) for an interesting discussion on the different ecological relations inside the host that could be synthesized in functions $\tilde{\beta }$ and $\tilde{\mu }$.

Let $\tilde{\beta }$ be such that

$$ \frac{d}{dx} \tilde{\beta }(0,x)=1, $$

which introduces a biological interpretation of the phenotype *x*: increasing the phenotype in one unit increases in one unit the pathogen replication rate in the absence of competition (i.e., at the infection onset V≈0). We only consider positive phenotypes, i.e. x>0, and assume that $$\tilde{\beta }(V,x)=x\beta (V)$$ with β(0)=1, β(V)>0 and $\beta^{\prime }\left(V\right)<0$ for all *V*, as well as the fact that β tends to vanish as *V* tends to infinity (i.e. $$\beta (V)\rightarrow 0$$ as $$V\rightarrow \infty $$). Similarly, we take $$\tilde{\mu }(V,x)=\phi (x)\mu (V)$$ with μ(0)=1, μ(V)>0 and $\mu^{\prime }\left(V\right)>0$ for all *V*, with ϕ being a positive function, so that Eq. (6) becomes

7 $$\begin{aligned} \dot{V} = \Gamma (V,x) V, \qquad \Gamma (V,x) = x\beta (V) -\phi (x)\mu (V). \end{aligned}$$

The term ϕ(x) reflects the collateral effects an increase in *x* (and, thus, an increase in the replication rate at the onset of the infection) may have on the elimination rate of the pathogen. For instance, a higher replication rate may entail viral capsules less stable (in case the pathogen is a virus), and hence an increase of the capsules’ natural degradation. For this reason we assume that function ϕ is increasing. This is a minimalist within-host model since we want it to represent a wide range of biological systems. If we were focusing on a particular pathogen-host relation, then we could consider more complicated models (such as, for example, Barik et al. (2022)).

Notice that the assumptions on functions β and μ (together with the fact that *x* and ϕ(x) are positive) imply that Γ(V,x) is a strictly decreasing function of *V* for all x>0 and that it is negative for large enough *V*. Biologically, this means that competition between pathogens is always stronger than any possible mutualistic effect between them (such as the formation of biofilms). Mathematically, this implies the existence of a global attractor to which the pathogen population converges (see Proposition 4 in the Appendix), which is the unique positive solution to Γ(V,x)=0 if Γ(0,x)>0 and 0 if $\Gamma (0,x)\le 0$. Let us refer to this attractor as $\bar{V}(x)$, which is given implicitly by

8 $$\begin{aligned} \frac{\beta (\bar{V}(x))}{\mu (\bar{V}(x))}=\frac{\phi (x)}{x} \qquad \text {when}\qquad \Gamma (0,x)>0 \end{aligned}$$

and it is 0 otherwise.

To study the pathogen evolution within the host, we extend Eq. (7) to a polymorphic population with *n* strains whose phenotypes are $x_1,x_2,\dots ,x_n$ and whose abundances are denoted by $V_1,V_2,\dots ,V_n$, respectively. Specifically we consider

9 $$\begin{aligned} \dot{V}_i = \Gamma (V, x_i) V_i \qquad \text {with}\qquad \Gamma (V, x_i) = x_i \beta (V) - \phi (x_i) \mu (V), \quad V=\sum _{i=1}^n V_i. \end{aligned}$$

Notice that we are assuming that the ecological interactions modeled through β and μ depend only on the total number of pathogens, *V*. In other words: the particularities of each strain cannot affect these functions. This leaves out situations in which, for instance, one strain is specially inflammatory and its presence in the system supposes an increase in the degradation rate of other strains.

The dynamics described by Eq. (9) has two distinguished phases. If *V* is low, as occurs at the beginning of the infection, pathogens do not compete, meaning that the growth rate of a pathogen population with phenotype $x_i$ is approximately $\Gamma (0,x_i)=x_i\beta (0) - \phi (x_i)\mu (0)=x_i-\phi (x_i)$. Moreover, the largest $\Gamma (0,x_i)$, the faster the population grows at the infection onset, so the fittest phenotype at this phase is given by the value *x* maximizing Γ(0,x).

At some point, pathogens start to compete between them. It can be shown (see Proposition 4 in the Appendix) that, if the ratios $R(x_1),R(x_2),\dots ,R(x_n)$ with

$$ R(x):=\frac{x}{\phi (x)} $$

are all different and there is at least one strain with R(x)>1, then all but one strain tend to disappear. The surviving strain is the one associated to the largest ratio *R*(*x*). That is, if $x_i$ is such that $R(x_i)>R(x_j)$ for all j≠i and $R(x_i) > 1$, then $V_i$ stabilizes at $\bar{V}(x_i)$ while all other populations vanish. Thus, for a mutant pathogen with phenotype *y* to proliferate and replace, in the long term, a resident population with phenotype *x*, *R*(*y*) must be larger than *R*(*x*). At this later phase, therefore, pathogens maximizing *R*(*x*) are selected.

Whereas Γ(0,x) is the growth rate of the pathogen population at the beginning of the infection, *R*(*x*) is the reproduction number of the pathogen: it gives the expected offspring of a primary pathogen that enters into the host (Cushing and Diekmann 2016). Therefore, both Γ(0,x) and *R*(*x*) have important biological implications: at low densities the population increases if Γ(0,x)>0 or R(x)>1 (and it decreases if Γ(0,x)<0 or R(x)<1). That the sign of Γ(0,x) coincides with the sign of R(x)-1 is established for general models in Thieme (2009). In our system this relation follows easily from the definitions:

10 $$\begin{aligned} \Gamma (0,x)=x-\phi (x)=\phi (x)\left( \frac{x}{\phi (x)}-1\right) =\phi (x)\left( R(x)-1\right) . \end{aligned}$$

There are thus two criteria to asses what is the optimal phenotype for a pathogen to grow more than its competitors within a host. Each of these criteria is meaningful at different infection phases. It turns out that it cannot exist a phenotype *x* which is optimal according to the two criteria. This follows easily from Eq. (10). Indeed, if *x* maximizes Γ(0,x) and *R*(*x*) simultaneously, then necessarily

$$ \frac{d}{dx}\Gamma (0,x)=0=R'(x)\quad \Rightarrow \quad R(x)=1 \quad \Rightarrow \quad \Gamma (0,x) = 0 , $$

which would imply that the pathogen is deemed to extinction no matter which phenotype it has. In fact, if we restrict to phenotypes for which R(x)>1 (i.e. phenotypes for which the pathogen population does not vanish), then (using that $\phi^{\prime }\left(x\right)>0$)

$$ \frac{d}{dx}\Gamma (0,x)\le 0\quad \Rightarrow \quad R'(x) \le -\frac{\phi '(x)}{\phi (x)}\left( R(x)-1\right) <0, $$

which means that if $$x_\text {r}$$ maximizes Γ(0,x), then the phenotype $$x_\text {k}$$ maximizing *R*(*x*) is necessarily smaller than $$x_\text {r}$$ (assuming that both Γ(0,x) and *R*(*x*) have only one local maximum). We use the notation $$x_\text {r}$$ and $$x_\text {k}$$ because adopting these phenotypes means, in the light of Remark 1 below, to follow an r-strategy or a k-strategy respectively, and the above observation shows that k-strategies are associated with lower replication rates than r-strategies. This makes totally sense if the host is interpreted as an ecosystem where a population is growing, since in this context r-strategists tend to degrade the environment more than k-strategists (because the later are selected to exploit the resources more efficiently whereas the former are selected to grow as fast as possible at the probable cost of overexploiting the media). The idea that individuals cannot thrive in both low and large population densities is not new (see Andrews and Harris (1986) for a good review on the topic). Verbal as well as quantitative population models have been used to explain why phenotypes selected in crowded habitats are somehow incompatible with those optimal at virgin environments. The premise underlying these models is that the replication rate positively correlates with the degradation rate (the more energy spent in reproduction, the less is spent in maintenance), and this premise is explicit in model (7) by taking $\phi^{\prime }\left(x\right)>0$.

### Remark 1

It turns out that maximizing function *R*(*x*) is equivalent to maximizing $$\bar{V}(x)$$ if $\beta^{\prime }\left(V\right)<0$ and $\mu^{\prime }\left(V\right)>0$. Indeed, from Eq. (8) it follows that the larger *R*(*x*) the smaller $$\beta (\bar{V}(x))/\mu (\bar{V}(x))$$, but

$$ \frac{d}{dV}\left( \frac{\beta (V)}{\mu (V)}\right) = \left( \frac{\beta '(V)\mu (V)-\beta (V)\mu '(V)}{\mu (V)^2}\right) <0, $$

so that the larger *R*(*x*) the larger $\bar{V}(x)$. Notice, however, that to prove Proposition 4 we just need Γ(V,x) to be a decreasing and eventually negative function of *V* (the monotonicity of β and μ do not play an essential role there). Whether the relation between *R* and $\bar{V}$ of this remark still holds under the assumptions of Proposition 4 is not addressed here.

## Linking The Two Models

Although it is clear that some mechanical relation must exist between the pathogen load inside the host and the parameters $k_1(x)$, $k_2(x)$ and $m_d(x)$ used in the epidemiological model, there is no natural formula to link them. The problem is that infected individuals in model (1) are not structured with respect to the age of infection, which means that the transmissibility of an infected individual is the same no matter for how long the pathogen has been growing in it (that is, regardless of the specific dynamics of the solution V(τ;x) of Eq. (6) with initial condition $V(0;x)=V_0$, where τ is the age of the infection and $V_0$ is the pathogen load at the infection onset). This issue could be solved by working with the density of infected individuals with respect to the age of infection (as it is done, for instance, in Gilchrist and Sasaki (2002); André and Gandon (2006); Pugliese (2011); Martcheva et al. (2017); Duan et al. (2023) and implicitly in Day (2001)). This would certainly establish a more natural relation between the within-host dynamics and the epidemiological parameters, at the cost of increasing the parameters’ dimensionality (the scalars $k_1(x)$, $k_2(x)$, $m_d(x)$ and *r* would become functions of the age of infection). To avoid this, and to make the analysis simpler, we stick to the non-structured epidemiological model and we link the epidemiological parameters with the within-host dynamics in a more phenomenological way.

Let us start by $k_1(x)$, which is the infection rate from resistant hosts that are infected with strain *x* (see Table 1). Resistant infected hosts leave their compartment at a neat rate of (m+r), either because they die from natural causes or because they recover from the infection. Thus, for them the duration of the infection is 1/(m+r). We assume that the rate $k_1(x)$ is proportional to the average pathogen load during this period of time. Notice that this is a simplification because transmission rate cannot arbitrarily grow as a function of the pathogen load since it is limited by the contact rate between hosts. Although a saturating function of the average pathogen load would be more realistic (see, for instance, Martcheva et al. (2017)), far from the saturated regime a linear relation between pathogen load and transmission rate is reasonable (Pugliese 2011; Ganusov et al. 2002) (as reasonable as assuming that doubling the pathogen load doubles the probability of infection once an infected has contacted a susceptible, which of course makes sense only if this probability is much lower than 1). We can reason analogously for the second infection rate $k_2(x)$ to get

11 $$\begin{aligned} k_1(x) \!=\! k \, (m\!+\!r) \int _0^{\frac{1}{m\!+\!r}} V(\tau ;x)d\tau , \quad k_2(x) = k \, (m+m_d(x)) \int _0^{\frac{1}{m+m_d(x)}}V(\tau ;x)d\tau , \end{aligned}$$

where *k* is a proportionality constant and V(τ;x) is the solution of

12 $$\begin{aligned} \left\{ \begin{array}{l} \dot{V} = x\beta (V)V -\phi (x)\mu (V)V\\ V(0;x)=1 \end{array} \right. . \end{aligned}$$

Notice that behind the initial condition in Eq. (12) there is the assumption that the pathogen load at the start of the infection of an individual is fixed (and equal to 1 if the units of the pathogen load are taken in relation to such a fixed initial load). This is a simplification (used in many within-host models, but see Gandolfi et al. (2015) for an exception) that allows us to proceed with the numerical analysis easily, although we are aware that some correlation between the pathogen load of the host that infects and the initial pathogen load of the infected host may exist in real systems.

Consider now $m_d(x)$, the mortality rate due to disease in vulnerable hosts infected with strain *x*. We assume it to be related to the velocity of pathogen replication because pathogen replication is the main process responsible for cellular damage in the host. The duration of infection in vulnerable hosts infected with strain *x* is $1/(m+m_d(x))$. Thus, in this case we assume that the rate $m_d(x)$ is proportional to the average replication velocity within the host during this period of time:

13 $$\begin{aligned} m_d(x) = c \, (m+m_d(x)) \int _0^{\frac{1}{m+m_d(x)}} x\beta (V(\tau ;x))V(\tau ;x)d\tau , \end{aligned}$$

where *c* is a proportionality constant and V(τ;x) is, again, the solution of Eq. (12). This is an implicit equation for $m_d(x)$. The relation between the induced mortality and the pathogen dynamics inside the host could be based on other mechanisms different to pathogen replication. Think of microbes releasing toxins or triggering a harmful inflammatory response. In these cases the pathogen load could be a better proxy of the harm experienced by the host (if pathogen load correlates with the toxin concentration or the immune response). This is why some models link the induced mortality with the pathogen load (Sasaki and Iwasa 1991) or consider within-host models in which the damage due to the immune response is explicitly taken into account (Pugliese 2011; Caudill and Lynch 2018). Host induced mortality has also been modeled as an all-or-nothing event in which the host is assumed to die due to the infection when the pathogen load surpasses a given threshold (Ganusov et al. 2002).

Let us remark that Eqs. (11) and (13) illustrate the complexity of considering co-infection events in the population model, Eq. (4). To obtain $k_1(x), k_2(x)$ and $m_d(x)$ we use the infection’s expected duration. This duration only depends on the host’s and pathogen’s physiology, and hence it is independent of $I(t;x) =(I_1(t;x),I_2(t;x))$ and $I(t;y)=(I_1(t;y), I_2(t;y))$. If co-infection were considered, however, we would need the co-infection’s expected duration within a host. But this quantity would no longer be independent of *I*(*t*; *x*), *I*(*t*; *y*). Indeed, co-infections tend to occur earlier if *I*(*t*; *x*) and *I*(*t*; *y*) are large, while they tend to occur later if *I*(*t*; *x*) and *I*(*t*; *y*) are small. We do not consider this scenario here, which amounts to assuming that the density of infected individuals is not too large.

## Consequences for the Evolution of Virulence

We showed that evolution will select the phenotype *x* maximizing the transmission potential *K*(*x*) (see Eq. (3)), which is a function of the phenotype-dependent parameters $k_1(x)$, $k_2(x)$ and $m_d(x)$, together with the natural mortality rate *m*, the recovery rate *r*, and the fraction of vulnerable hosts in the population, *p*. Then we linked a within-host dynamics with the population dynamics to show how the parameters $k_1(x)$, $k_2(x)$ and $m_d(x)$ might be estimated from the within-host dynamics.

This already provides a way to predict the selected value of any property dependent on the phenotype for a fixed choice of the other parameters. What we need to do is compute the optimal phenotype $x_\text {op}$, that is, the phenotype that maximizes *K*(*x*). As we will show later, for some parameter choices, the function *K*(*x*) has local and global maxima. Both of them might be relevant biologically: the local maximum can be attained when pathogen evolution is such that only small mutations are possible, whereas if mutations could cause large variations, then pathogens would inevitably evolve towards the phenotype corresponding to the global maximum of *K*.

We should keep in mind that for a strain *x* to be selected by evolution, it not only has to maximize *K*, but also has to fulfill bK(x)>m (otherwise, the virus disappears as time goes on, as we discussed earlier). The transmission potential *K*(*x*) already depends on *m*, but not on *b*. Thus, when studying properties of the system that depend on the selected phenotype, we also have to take into account the value of *b*, which could restrict the range of admissible phenotypes. In what follows, we present the results obtained when maximizing *K*(*x*) regardless of *b*, because any phenotype *x* could become admissible just by increasing *b*.

### Selected transmission potential

As commented earlier in Section 2, the transmission potential defined in Eq. (3) determines if the pathogen population persists (if K(x)>m/b) or extinguishes (if K(x)≤m/b).

**Fig. 1:**
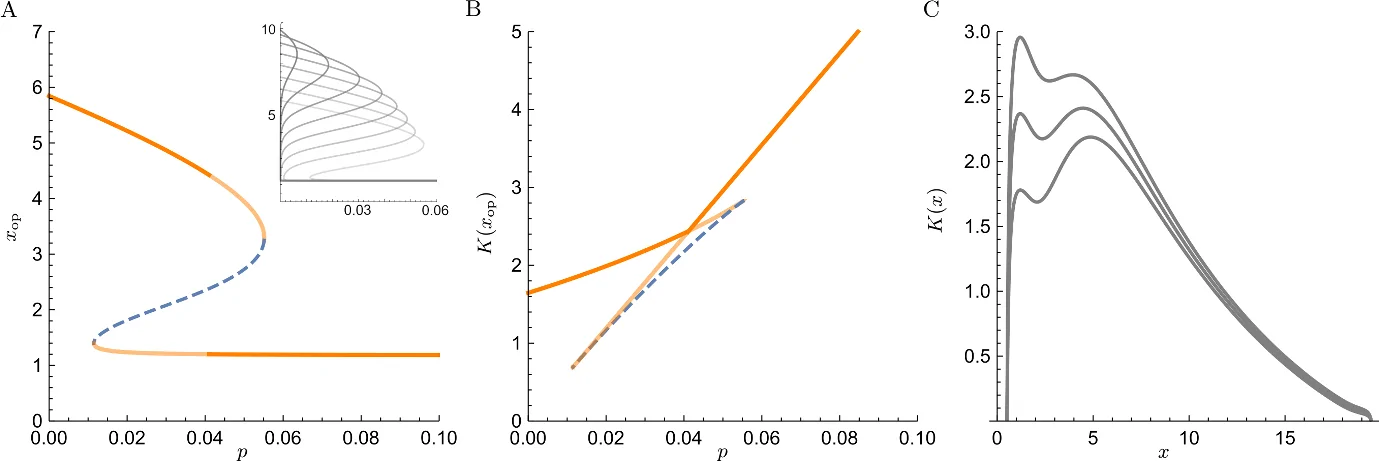
Selected phenotype $x_\text {op}$ as a function of *p* (in A) and selected transmission potential $K(x_\text {op})$ as a function of *p* on these phenotypes (in B), with *K* defined by Eq. (3). Here $x_\text {op}$ is the phenotype that globally (dark orange line) or locally (light orange line) maximizes *K*(*x*). The blue dashed lines give, as a function of *p*, the phenotype that locally minimizes *K*(*x*) (in A) and its corresponding transmission potential (in B). In C, functions *K*(*x*) are represented for p=0.02 (bottom curve), p=0.03 (middle curve) and p=0.04 (upper curve). Parameters: m=0.001, r=0.2. Functions of Eq. (6): $\tilde{\beta }(x,V)=x/(1+V/1000)$ and $\tilde{\mu }(x,V)=(10+x^2)/20$. Proportionality constants of Eqs. (11) and (13): k=0.0004 and c=0.00001. The inset in A shows the curves $x_\text {op}(p)$ for different choices of $\tilde{\beta }$ and *c*, specifically for $\tilde{\beta }(x,V)=x/(1+V/10^j)$ and $c=10^{-(j+2)}$ with $j\in \{3,4,5,6,7,8,9,10\}$ (the darker the larger *k*) (color figure online)

**Fig. 2:**
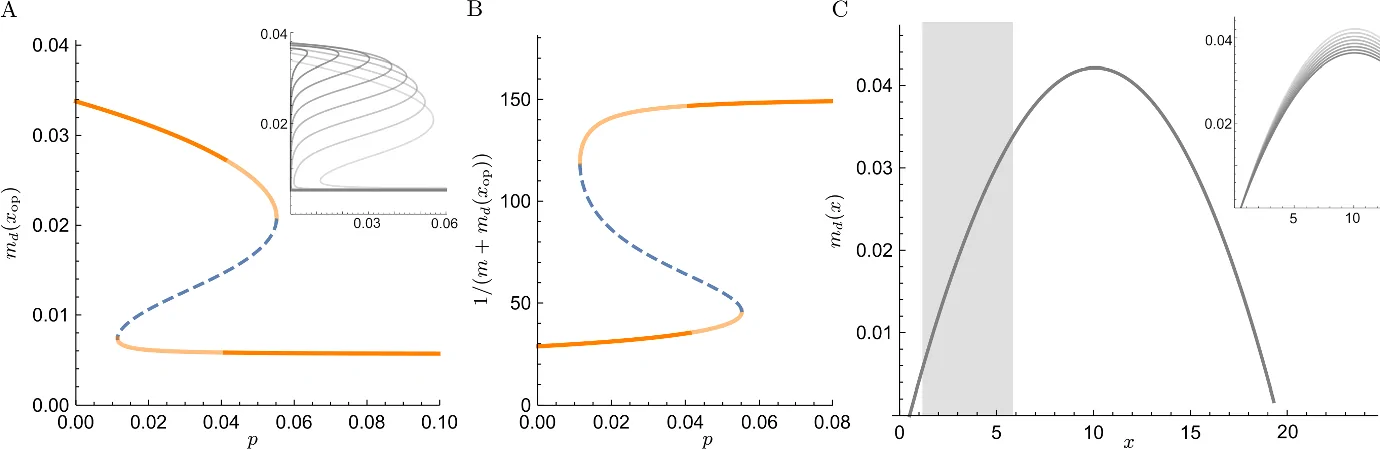
Selected pathogen virulence $m_d(x_\text {op})$ as a function of *p* (in A) and the corresponding life expectancy of vulnerable individuals (in B), computed as $1/(m+m_d(x_\text {op}))$. Here $x_\text {op}$ is the phenotype that globally (dark orange line) or locally (light orange line) maximizes the transmission potential *K*(*x*). The blue dashed lines indicate the virulence (in A) and the life expectancy (in B) associated to the phenotype that locally minimizes *K*(*x*). In C, function $m_d(x)$ is represented for the parameters used, with the gray band representing the range of *x* in Figure 1A (the range between the selected phenotype when p=1 and the selected phenotype when p=0). The relative linearity of function $m_d$ within this range explains the similarity of curves in 2A and Figure 1A. The insets in A and C are analogous to the one in Figure 1A: they show how the curve on the respective plot changes when $\tilde{\beta }$ and *c* are modified. Same parameters as in Figure 1 (color figure online)

In Figure 1A we show what is the locally optimal phenotype $x_\text {op}$ as a function of *p*. The dark and light orange curves correspond *K*(*x*) having a global or just a local maximum at $x_\text {op}$, respectively. Figure 1B shows the transmission potential once the virus has evolved towards such an optimal phenotype: for each *p*, the dark orange curve represents the global maximum of *K*(*x*) while the light orange curve represents a local maximum of *K*(*x*). In Figure 1B, the smooth orange curve with a lower slope corresponds to the local maximum associated with larger values of *x* (the upper orange branch of Figure 1A), whereas the smooth orange curve with a larger slope corresponds to the local maximum with lower values of *x* (the bottom orange branch of Figure 1A). We used a mortality rate m=0.001 (i. e., the natural lifespan of individuals is of 1000 days) and a recovery rate of r=0.2 (i. e., after 5 days from becoming infected a resistant individual is no longer infectious). These parameters could correspond, for instance, to a rodent species such as the house mouse. The pathogen degradation rate inside the host is taken to increase with *x* and to be independent of *V*, specifically $\tilde{\mu }(x,V)=(10+x^2)/20$, whereas we used a pathogen replication rate that decreases with *V* and increases linearly with *x*, specifically $\tilde{\beta }(x,V)=x/(1+c V/10^2)$, where the constant *c* is the same as in Eq. (13). By making $\tilde{\beta }$ to depend on *c* in such a way, the product $c \bar{V}(x)$ turns out to be independent on *c*, which allows us to analyze the effect of increasing the pathogen load at equilibrium (by decreasing *c*) without changing the order of magnitude of the induced mortality, i.e. $m_d(x)$ (the inset in Figure 2C shows that this is the case).

If mutations can induce large changes on the phenotype *x*, the selected transmission potential is simply the global maximum of function *K* for each *p* (represented by the dark orange curve in Figure 1A). Alternatively, if mutations have only small effects on *x*, then the selected transmission potential follows one of the local maxima of *K* as *p* varies. Notice that in this later case (taking the parameters used in Figure 1 and assuming that b/m=1, i. e., that the population variables are normalized so that they are relative to the population equilibrium in the absence of pathogens) the pathogen population eventually disappears if *p* decreases slowly while the pathogen’s phenotype is trapped in the local maximum associated with low values of *x* (i.e. if the transmission potential changes as a function of *p* following the orange curve in 1B with a larger slope). Indeed, as soon as the transmission potential at such a local maximum is lower than 1, the pathogen population cannot persist. Somehow paradoxically, however, if *p* decreases abruptly and becomes very small (close enough to zero so that there are not two local maxima anymore), then the pathogen is able to evolve towards the maximum associated to large values of *x*, for which the transmission potential is larger than 1. On the other hand, if the phenotype is trapped in the local maximum associated with large values of *x*, then the pathogen persists for any value of *p*. If *p* becomes large enough, however, this maximum disappears and the phenotype shifts towards the maximum associated with low values of *x*.

#### Remark 2

The evolutionary landscape depicted by function *K* can be interpreted within the adaptive dynamics framework (Geritz et al. 1998; Kisdi 2020). The invasion fitness of a mutant pathogen with trait *y* that appears within a resident population of trait *x* in equilibrium (using $m_1=m+r$, $m_2(y)=m+m_d(y)$ and q=1-p to make the expression more concise) is

$$ s(x,y) = \frac{1}{2}\left( a+\sqrt{a^2+4 m_1 m_2(y)\left( \frac{K(y)}{K(x)}-1\right) }\right) $$

with

$$ a=(m_1+m_2(y))\left( \frac{K(y)}{K(x)}-1\right) -\frac{1}{K(x)}\left( q k_1(y)\frac{m_2(y)}{m_1}+p k_2(y)\frac{m_1}{m_2(y)}\right) . $$

Function *s*(*x*, *y*) gives the growth rate of the mutant population and it is obtained by linearizing system (4) at the equilibrium $(\bar{S}(x),\bar{I}_1(x),\bar{I}_2(x),0,0)$) detailed in Eq. (5). Notice that, in agreement with Proposition 3, if K(y)<K(x) then s(x,y)<0 (because a<0 and the radicand is smaller than $a^2$ in this case), whereas if K(y)>K(x) then s(x,y)>0 (because the radicand is larger than $a^2$ in this case). Function *s*(*x*, *y*) could be analyzed following the adaptive dynamic framework to obtain singular evolutionary strategies. The analysis would identify the local maxima of *K* with evolutionary attractors and the local minima of *K* (when it exists) with an evolutionary repellor. However, such an analysis is redundant if Proposition 3 is taken into account.

### Selected virulence

Now we study the effect of the immune response on the selected virulence. The virulence of a strain *x* is defined as the parameter $m_d(x)$ itself. This is a natural choice since the virulence is generally defined as a measure of the harm produced by the virus to its host, and $m_d(x)$ is the mortality rate caused by a virus with phenotype *x*. Thus, the selected virulence is $m_d(x_\text {op})$.

Figure 2 shows the selected virulence $m_d(x_\text {op})$ as a function of *p*. As in Figure 1, we plot the selected virulence for both slightly and highly mutable viruses (corresponding to the phenotypes at which *K*(*x*) is locally and globally maximal, respectively).

The first thing to notice is that the fraction of immunocompromised hosts, *p*, has an important effect on the selected virulence. In the extreme case of a host population composed solely of resistant individuals (p=0), evolution will select highly virulent strains. On the contrary, in a host population with only vulnerable individuals (p=1), viruses with a very low virulence will prevail. Figure 2B shows that the life expectancy of an infected vulnerable individual in the low virulence regime (the virulence selected when p=1) is almost 6 times the life expectancy in the high virulence regime (the virulence selected when p=0).

If viruses have a high capacity to mutate (mutations may change the virulence a lot), the selected virulence follows the dark orange curve shown in Figure 2A. This means that virulence decreases as *p* increases, but not continuously: there is a critical value of *p* at which the virulence suddenly drops. In the vicinity of this value, small variations in *p* can lead to very different outcomes in terms of the selected virulence.

If, on the contrary, viruses have only a limited capacity to mutate (mutations slightly change the virulence), the selected virulence will follow the solid orange lines in the same diagram (either the upper branch or the lower branch). In particular, there is an intermediate range of *p* for which two different outcomes (characterized by a low and a high virulence level) are possible. The selected virulence in this case shows hysteresis: there is a range of *p* in which the selected virulence depends on the virulence of the strain currently present in the system. The inset in Figure 2A shows that this range tends to shrink and shift towards lower values of *p* as the pathogen load at equilibrium increases while maintaining the induced mortality as a function of *x* relatively similar to that shown in Figure 2C (this is accomplished by reducing parameter *c* in a specific way as commented above). Notice, however, that the selected low and high virulences in the hysteresis curves are roughly the same.

Another consequence of this phenomenon is that, if *p* changed slowly with time (for example due to vaccination campaigns or because hosts become more resistant due to an improvement of the environment they inhabit), the selected virulence would depend on what was the virulence in the past. Going from large *p* to low *p* would only result in a high virulence being selected if *p* drops below very low values (around 0.01 in this particular example). On the contrary, going from low *p* to large *p* would maintain large virulence levels unless *p* becomes large enough (around 0.055 in the example).

**Fig. 3:**
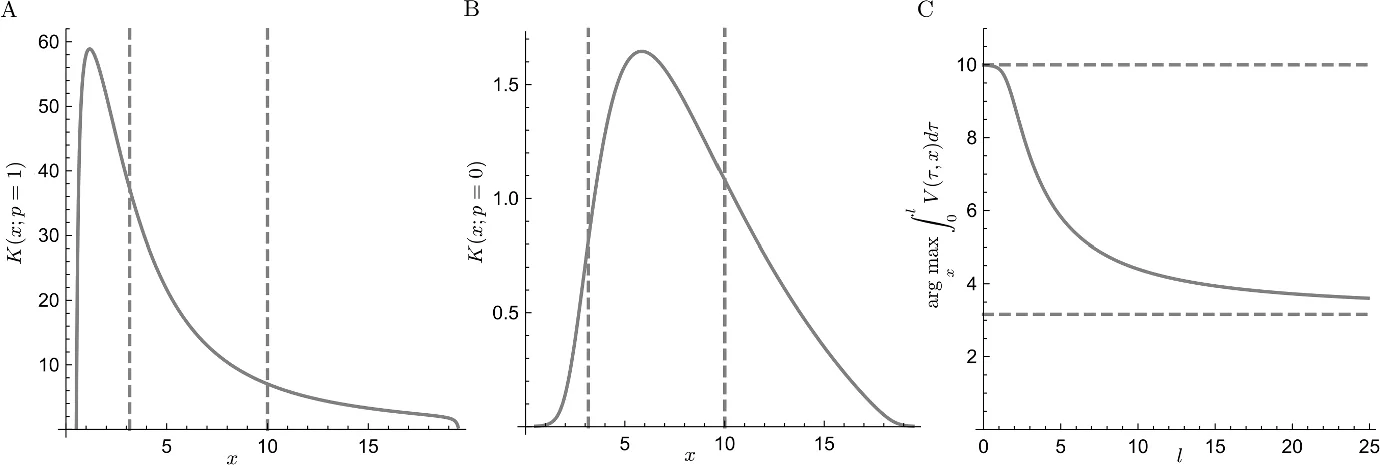
Transmissibility of vulnerable (A) and resistant (B) individuals. The former transmit much more than the later due to larger infection lifetimes ($(m+m_d(x))^{-1}>(m+r)^{-1}$) and higher pathogen loads ($k_2(x)>k_1(x)$). The dashed lines correspond to the phenotypes maximizing R(x)=x/ϕ(x) (left line) and $\Gamma (x,0)=x-\phi (x)$ (right line) introduced in Section 3 (recall Remark 1 stating that maximizing *R*(*x*) is the same as maximizing $\bar{V}(x)$). In C, the phenotype maximizing the integral $\int _0^l V(\tau ,x)d\tau$ with *V* being the solution of Eq. (12) is represented as a function of *l* (the infection lifetime). The dashed lines represent the same as in A and B. Same parameters as in Figure 1

It is worth noticing that virulence barely changes as a function of *p* when it evolves along the low virulence regime (i.e. the lower curve in Figure 2A is essentially constant and equal to the selected virulence when p=1). What happens is that the selection pressure acting on the pathogens is mostly due to vulnerable individuals if they are not too rare and *x* is small. In this situation most transmissions are caused by vulnerable individuals (it does not matter if there are immunocompetent individuals in the population because the transmissions caused by them are very low in comparison), and this is why the selected virulence in this regime is essentially the one that maximizes the transmissibility of vulnerable individuals. To understand this more clearly, Figure 3 shows that the capacity of vulnerable individuals to transmit the pathogen is considerably larger than that of resistant individuals, i.e. that

14 $$\begin{aligned} \frac{k_2(x)}{m+m_d(x)} \gg \frac{k_1(x)}{m+r}, \end{aligned}$$

and hence maximizing *K*(*x*) is almost the same as maximizing the left hand side of Eq. (14) if *p* is not too small.

In Figure 3C it is shown what is the phenotype maximizing the accumulated pathogen load within the host as a function of the infection lifetime, denoted by *l*. This pattern is coherent with the observations made in Section 3. Indeed, if *l* is small, then the replication of the pathogen is mostly on the exponential phase and hence the replication rate Γ(x,0) is maximized ($x_\text {r}$ is selected). Alternatively, if *l* is very large, then the pathogen population is mostly on the stationary phase, and hence $\bar{V}(x)$ (or *R*(*x*) in the light of Remark 1) is maximized ($x_\text {k}$ is selected). For intermediate values of *l*, the selected phenotype lies between $x_\text {r}$ and $x_\text {k}$.

Since recovery times of immunocompetent individuals are short, one would expect the selected phenotype when p=0 to be close to $x_\text {r}$ (i.e. the maximum of the curve in Figure 3B to be close to the right dashed line). For the parameters we are considering, however, it turns out that 1/(m+r)≈5 days is not so short (the growth of the pathogen population has already slowed down considerably due to competition when the immune system terminates the infection). Analogously, if the induced mortality of the pathogen is small compared to the recovery rate, then it seems that the selected pathogen when p=1 should be close to $x_\text {k}$ (i.e. the maximum of the curve in Figure 3A should be close to the left dashed line). What explains the discrepancy in this case is the fact that the induced mortality is also affected by natural selection, since transmission is affected by both the accumulation of pathogens throughout the infection as well as the infection lifetime itself (i.e. $1/(m+m_d(x))$). By reducing *x* beyond $x_\text {k}$ both the pathogen load at equilibrium (i.e. $\bar{V}(x)$) and the induced mortality rate (i.e. $m_d(x)$) diminish, and this can occur as long as the benefit of the later surpasses the detriment of the former.

#### Remark 3

Although the evolutionary outcome discussed in this section depends on a particular choice for functions $\tilde{\beta }$ and $\tilde{\mu }$, qualitatively analogous results hold provided that

15 $$\begin{aligned} \frac{K''(x;p=1)}{K'(x;p=1)} < \frac{K''(x;p=0)}{K'(x;p=0)}\quad \text {for some}\quad x\in (x_\text {op}(1),x_\text {op}(0)), \end{aligned}$$

where *K*(*x*; *p*) is the transmission potential given in Eq. (3) and $$x_\text {op}(0)$$ and $$x_\text {op}(1)$$ are the phenotypes maximizing *K* when p=0 and p=1, respectively (we assume that *K* has only one maximum when p=0 or p=1 and that $$x_\text {op}(0)>x_\text {op}(1)$$). Indeed, the discussion is based on the hysteresis dynamics shown in Figs. 1 and 2, and it turns out that the hysteresis exists if and only if the previous condition is satisfied. To see this, recall that the curve in Fig. 1A gives phenotypes for which $K^{\prime }\left(x;p\right)=0$, and notice that $K^{\prime }\left(x;p\right)=\left(1-p\right)K^{\prime }\left(x;p=0\right)+pK^{\prime }\left(x;p=1\right)$. Therefore, the curve $K^{\prime }\left(x;p\right)=0$ can be given as the graph of the function

$$ p(x) = \frac{K'(x;p=0)}{K'(x;p=0)-K'(x;p=1)}, $$

so the hysteresis exists if and only if $p^{\prime }\left(x\right)>0$ for some $$x\in (x_\text {op}(1),x_\text {op}(0))$$, and this condition is equivalent to Eq. (15). Since K(x;p=0) and K(x;p=1) depend on $$\tilde{\beta }(x,V)=x\beta (V)$$ and $$\tilde{\mu }=\phi (x)\mu (V)$$ through Eqs. (11), (12) and (13), the above condition translates into a condition for the within-host functional responses β, ϕ and μ. However, in the condition the role of these functions is not explicit but indirect through the effects they have on function *V*(*t*; *x*).

## Discussion

In this work we have shown that pathogens infecting heterogeneous populations of hosts evolve in a fitness landscape with multiple hills, meaning that pathogens can evolve towards different virulence values depending on the initial phenotype they have (provided that mutations have small effects on the phenotype). This draws some interesting consequences when the heterogeneity reflects the immunity level of the hosts. The simple dimorphic population we considered makes the fitness landscape have two local peaks under certain conditions. This occurs when it is better for the pathogen to specialize in one of the hosts instead of adopting a generalist strategy to explode both of them in a balanced way. Slightly changing the proportions of host types barely modifies the position of the peaks. Large changes in the structure of the host population, however, may cause the hosts in which pathogens are specialized be too rare, in the sense that it is not worth it for them to keep being specialized in such an infrequent host type. When this happens, natural selection favors the pathogen to explode the other host type more efficiently, i.e., the virulence shifts and the pathogen becomes specialized in the dominant host type. In particular, if for whatever reason the fraction of vulnerable hosts decreases beyond a given point, the lethality caused by the pathogen on immunocompromised individuals can increase considerably. It is as if being weak and uncommon made you even weaker that what you would be if the ratio of weak individuals were higher. We refer to this indirect interaction as the “weakening of the uncommon weak”.

As it was suggested in Sorci et al. (2013), immunocompromised individuals favor the emergence of many diseases by allowing the pathogen to grow. The observations drawn in the model presented here, however, point towards a different effect of the immune system on virulence evolution: it is the combination of both resistant and immunocompromised individuals what can make the pathogen be fatal for the later. Although it is not easy to asses if this phenomenon really occurs in the wild, we can think of some situations in which it could be considered at least as a possibility. The collapse of bee colonies is interesting in this regard. Cases of abandoned and dying hives have been reported since the 2000s. Initially it was assumed that a highly virulent emergent pathogen was the cause behind them, but different studies showed that diseased colonies were affected by viruses or parasites known to infect bees in the past in an asymptomatic way (Paxton 2010; Ratnieks and Carreck 2010). The virulence of these pathogens seems to have increased dramatically after the bees’ immune response impaired due to a second agent, such as another parasite (the most important being the mite *Varroa destructor*) or contaminants (insecticides used in crops).

The model presented here is a proof of concept on what could happen in the wild. Further work should address to what extent the multiplicity of locally optimal virulences is preserved when the structure of the host population is more complex. While there are different studies in which the effects of heterogeneity on the evolution of virulence are assessed, some of them pointing that host heterogeneity favors the selection of higher virulence values and some others concluding the opposite (Ganusov et al. 2002; Pugliese 2011), less attention has been paid to the existence of multiple local evolutionary stable strategies (ESS). In Regoes et al. (2000) a dimorphic host population is also considered and a trade-off is assumed on pathogens preventing them to specialize in both host types: the higher the virulence and infectivity in one host, the lower in the other. If such a trade-off penalizes being a generalist, then the virulence fitness landscape has two peaks, corresponding to the virulences specialized to each host type. A similar result in this direction was obtained in Gandon (2004), in which evolutionary branching is shown to occur if interspecific transmission is small compared to intraspecific transmission. In this situation pathogens first evolve towards a generalist phenotype, but from there selection favors pathogen specialization to the different hosts (since competition between generalist pathogens is strong enough to favor specialization). This is, however, a different mechanism by which pathogens can diversify, based on pathogen-host affinities and not on the intensity of the hosts’ immune responses. The model presented in André and Gandon (2006) is much closer to ours in this regard. There, vaccinated and non-vaccinated hosts are considered (the analogues of our immunocompetent and vulnerable hosts), and the bistability of pathogen virulences is also observed despite some conceptual differences with our model. In Osnas et al. (2015) the bistability is also observed in a two-species setting, and it is shown that the set of parameters associated to bistability increases if interspecific transmissions decrease. Since the model used by Osnas et al. is similar to that used in Gandon (2004), the discrepancy in their respective evolutionary outcomes is striking. Osnas et al. argue that the reason why in their model two (monomorphic) ESS coexist whereas in Gandon’s model evolution leads towards a dimorphic pathogen population is unclear, although they point to indirect effects caused by the frequency-dependent transmission rate they use. In fact, a model resembling the ones just mentioned is used in Cousineau and Alizon (2014) to analyze virulence evolution in a sex-based heterogeneous population, but there, only one ESS is found for a large range of parameters (and evolutionary branching is possible only in extreme cases). These observations suggest that the system details may have a significant effect on virulence evolution. In this regard, it is worth emphasizing that the model presented here differs from the ones above in the following: it only considers heterogeneous infected individuals (and hence the susceptible class is a one-dimensional variable). This simplified structure is what allows us to give global results on the dynamics, an issue that was not addressed in the other works.

More realistic human oriented epidemiolgical models include the capacity of the health system to diagnose and treat infected individuals by assuming that the recovery rate saturates (Gion and Saito 2023). Analyzing in which direction this factor affects the results presented here could shed some light on how pathogen virulence evolves depending on the resources allocated to the health system.

## Data Availability

Not applicable.

## Appendix: Global Stability Analysis

### Proposition 1

Consider the system

A1 $$\begin{aligned} \begin{aligned} S'(t) =&\, b - m S (t) - \left( k_1 I_1 (t)+k_2 I_2 (t)\right) S (t) \\ I_1'(t) =&\, (1-p) \left( k_1 I_1 (t)+k_2 I_2 (t)\right) S (t) - m I_1(t) - r I_1(t)\\ I_2'(t) =&\, p \left( k_1 I_1 (t)+k_2 I_2 (t)\right) S (t) - m I_2(t) - m_d I_2(t) . \end{aligned} \end{aligned}$$

If the quantity

$$ K:=(1-p)\frac{k_1}{m+r}+p\frac{k_2}{m+m_d} $$

satisfies bK≤m, then system (A1) has a disease-free equilibrium $\bar{P}_0 = (\bar{S}_0, 0, 0)$ with

A2 $$\begin{aligned} \bar{S}_0 = \frac{b}{m} \end{aligned}$$

and it is globally asymptotically stable in the region

$$ \Omega := \{ (S,I_1,I_2) \,:\, S, I_1, I_2 \ge 0\}. $$

### Proof

Let $S^* = 1/K$. We notice that $S^* \ge \bar{S}_0$ because bK≤m. In Ω, if $S > S^*$, then

A3 $$\begin{aligned} \dot{S} = b - m S - (k_1 I_1 + k_2 I_2) S < b-m S^* \le b-m \bar{S}_0 = 0. \end{aligned}$$

In addition, from Eq. (A1) it also follows

A4 $$\begin{aligned} (S+I_1+I_2)'\le b - m(S+I_1+I_2). \end{aligned}$$

Thus, Eqs. (A3) and (A4) imply that any orbit in Ω enters or approaches asymptotically the positively invariant compact region

$$ \tilde{\Omega } := \Omega \cap \{ (S,I_1,I_2) \,:\, S \le S^*\quad \text {and}\quad S+I_1+I_2 \le b/m \}, $$

and hence it is enough to show that all orbits starting in $\tilde{\Omega }$ converge to $\bar{P}_0$ (since orbits are continuous with respect to initial conditions).

Within $\tilde{\Omega }$, we consider the Lyapunov function

A5 $$\begin{aligned} V(S,I_1,I_2) = \tau _1 I_1 + \tau _2 I_2 \end{aligned}$$

where $\tau _1, \tau _2$ are constants to be determined.

Clearly, *V* has a global minimum at $\bar{P}_0 = (\bar{S}_0, 0, 0)$. In the next paragraph we prove that

$$ \frac{\text {d}}{\text {d}t} V(S(t),I_1(t),I_2(t)) \le 0 \quad \forall t $$

and that the only invariant subset of $\{\frac{\text {d}}{\text {d}t} V(S(t),I_1(t),I_2(t)) = 0\}$ is the set $\{I_1=0, I_2=0\}$. Then, by LaSalle’s invariance principle we have that all accumulation points of orbits in $\tilde{\Omega }$ belong to the set $\{I_1=0, I_2=0\}$. Since all orbits in this set converge to $\bar{P}_0$, we conclude that all orbits of $\tilde{\Omega }$ (and hence Ω) converge to $\bar{P}_0$.

On the one hand, differenciating $V = V(S(t),I_1(t),I_2(t))$ with respect to time and defining $m_1:= m+r$, $m_2:= m+m_d$, we have

A6 $$\begin{aligned} \begin{array}{lll} \dot{V} & =& \displaystyle \tau _1 \dot{I}_1 + \tau _2 \dot{I}_2 \\ & =& \displaystyle \tau _1 \left[ (1-p) \left( k_1 I_1 +k_2 I_2\right) S - m_1 I_1 \right] + \tau _2 \left[ p \left( k_1 I_1 +k_2 I_2 \right) S - m_2 I_2 \right] \\ \\ & =& \displaystyle \tau _1 m_1 \left[ \left( \frac{(1-p) k_1}{m_1} + \frac{\tau _2 k_1 m_2}{\tau _1 k_2 m_1} \frac{p k_2}{m_2} \right) S - 1 \right] I_1 \\ & & \qquad \qquad + \tau _2 m_2 \left[ \left( \frac{\tau _1 k_2 m_1}{\tau _2 k_1 m_2} \frac{(1-p) k_1}{m_1} + \frac{p k_2}{m_2} \right) S - 1 \right] I_2. \end{array} \end{aligned}$$

Now, taking $\tau _1, \tau _2$ such that

A7 $$\begin{aligned} \tau _2 k_1 m_2 = \tau _1 k_2 m_1, \end{aligned}$$

we obtain

A8 $$\begin{aligned} \begin{array}{lll} \dot{V} & \!=\!& \displaystyle \tau _1 m_1 \left[ \left( \frac{(1\!-\!p) k_1}{m_1} \!+\! \frac{p k_2}{m_2} \right) S \!-\! 1 \right] I_1 \!+\! \tau _2 m_2 \left[ \left( \frac{(1-p) k_1}{m_1} \!+\! \frac{p k_2}{m_2} \right) S - 1 \right] I_2 \\ \\ & =& \displaystyle \left( K S - 1 \right) \left( \tau _1 m_1 I_1 + \tau _2 m_2 I_2 \right) \\ & \le & \displaystyle \left( K S^* - 1 \right) \left( \tau _1 m_1 I_1 + \tau _2 m_2 I_2 \right) \\ & =& 0. \end{array} \end{aligned}$$

On the other hand, using Eq. (A8), we see that $$\dot{V}=0$$ in $$\tilde{\Omega }$$ implies $$I_1 = I_2 = 0$$ or $$S=1/K=S^*$$, and clearly the only invariant set in $$\{I_1 = I_2 = 0\}\cup \{S=S^*\}$$ is the set $$\{I_1 = I_2 = 0\}$$, since $S^{\prime }<0$ on $$S^*$$ if $$I_1>0$$ or $$I_2>0$$. □

### Proposition 2

Consider the system

A9 $$\begin{aligned} \begin{aligned} S'(t) =&\, b - m S (t) - \left( k_1 I_1 (t)+k_2 I_2 (t)\right) S (t) \\ I_1'(t) =&\, (1-p) \left( k_1 I_1 (t)+k_2 I_2 (t)\right) S (t) - m I_1(t) - r I_1(t)\\ I_2'(t) =&\, p \left( k_1 I_1 (t)+k_2 I_2 (t)\right) S (t) - m I_2(t) - m_d I_2(t) . \end{aligned} \end{aligned}$$

If the quantity

$$ K:=(1-p)\frac{k_1}{m+r}+p\frac{k_2}{m+m_d} $$

satisfies bK>m, then system (A9) has an endemic equilibrium $\bar{P} = (\bar{S}, \bar{I}_1, \bar{I}_2)$ with

A10 $$\begin{aligned} \bar{S} = 1/K, \qquad \bar{I}_1 = \frac{1-p}{m+r} \left( b - \frac{m}{K} \right) , \qquad \bar{I}_2 = \frac{p}{m+m_d} \left( b - \frac{m}{K} \right) , \end{aligned}$$

and it is globally asymptotically stable in the positive cone

$$ \mathring{\Omega } = \{(S,I_1,I_2) \in \mathbb {R}^3 \,:\, S, I_1, I_2 > 0 \}. $$

### Proof

Following Guo et al. (2012), we consider the Lyapunov function in $\mathring{\Omega }$

A11 $$\begin{aligned} V(S,I_1,I_2) = \tau _0( S - \bar{S} \log S ) + \tau _1( I_1 - \bar{I_1} \log I_1 ) + \tau _2( I_2 - \bar{I_2} \log I_2 ), \end{aligned}$$

where $\tau _0, \tau _1, \tau _2$ are positive constants to be determined.

Clearly, *V* has a **unique** global minimum at $\bar{P} = (\bar{S}, \bar{I}_1, \bar{I}_2)$. We have to prove that, in $\mathring{\Omega }$,

$$ \dot{V} = \frac{\text {d}}{\text {d}t} V(S(t),I_1(t),I_2(t)) \le 0 \quad \forall t $$

and that the only invariant set within the set $\mathring{\Omega } \cap \{\dot{V}= 0\}$ is the singleton $\{\bar{P}\}$. Then we will have, by LaSalle’s invariance principle, that $\{\bar{P}\}$ is globally asymptotically stable in the positive cone $\mathring{\Omega }$.

Let us introduce the auxiliary variable

A12 $$\begin{aligned} I_0(t) := \left( k_1 I_1 (t)+k_2 I_2 (t)\right) S (t), \end{aligned}$$

and let $\bar{I}_0$ refer to the associated equilibrium:

A13 $$\begin{aligned} \bar{I}_0 = \left( k_1 \bar{I}_1+k_2 \bar{I}_2\right) \bar{S} = b-\frac{m}{K}. \end{aligned}$$

Differenciating $V = V(S(t),I_1(t),I_2(t))$ with respect to time we have

A14 $$\begin{aligned} \begin{array}{lll} \dot{V} & =& \displaystyle \frac{\partial V}{\partial S} \dot{S} +\frac{\partial V}{\partial I_1} \dot{I}_1 +\frac{\partial V}{\partial I_2} \dot{I}_2 \\ \\ & =& \displaystyle \tau _0 \left( 1 - \frac{\bar{S}}{S} \right) \left( b - m S - I_0 \right) \\ & & \displaystyle + \tau _1 \left( 1 - \frac{\bar{I}_1}{I_1} \right) ( (1-p) I_0 - (m+r) I_1 ) \\ & & \displaystyle + \tau _2 \left( 1 - \frac{\bar{I}_2}{I_2} \right) ( p I_0 - (m+m_d) I_2 ) \\ \\ & =& \tau _0 T_0 + \tau _1 T_1 + \tau _2 T_2. \end{array} \end{aligned}$$

We analyze the terms $T_0, T_1, T_2$ accompanying $\tau _0$, $\tau _1$ and $\tau _2$ separately. Using the fixed point equations (A10), (A13) we have

A15 $$\begin{aligned} \begin{array}{lll} T_0 & =& \displaystyle - \frac{m}{S} (S-\bar{S})^2 + \sum \limits _{j=1}^2 k_j \bar{S} \bar{I}_j \left( 2 - \frac{\bar{S}}{S} - \frac{I_0}{\bar{I}_0} + \frac{I_j}{\bar{I}_j} - \frac{S \bar{I}_0 I_j}{\bar{S} I_0 \bar{I}_j} \right) \\ T_1 & =& \displaystyle (1-p) \bar{I}_0 \left( 1 + \frac{I_0}{\bar{I}_0} - \frac{I_1}{\bar{I}_1} - \frac{I_0 \bar{I}_1}{\bar{I}_0 I_1} \right) \\ T_2 & =& \displaystyle p \bar{I}_0 \left( 1 + \frac{I_0}{\bar{I}_0} - \frac{I_2}{\bar{I}_2} - \frac{I_0 \bar{I}_2}{\bar{I}_0 I_2} \right) . \end{array} \end{aligned}$$

We notice that, for $j \in \{1,2\}$,

A16 $$\begin{aligned} 1 + \frac{I_0}{\bar{I}_0} - \frac{I_j}{\bar{I}_j} - \frac{I_0 \bar{I}_j}{\bar{I}_0 I_j} = \left( 1 - \frac{I_0 \bar{I}_j}{\bar{I}_0 I_j} + \log \frac{I_0 \bar{I}_j}{\bar{I}_0 I_j}\right) + \left( \frac{I_0}{\bar{I}_0} - \log \frac{I_0}{\bar{I}_0} - \frac{I_j}{\bar{I}_j} + \log \frac{I_j}{\bar{I}_j} \right) , \end{aligned}$$

and the term under the first parenthesis is non-positive because $1-x+\log x \le 0$ for x>0.

Similarly,

A17 $$\begin{aligned} \begin{aligned} 2 - \frac{\bar{S}}{S} - \frac{I_0}{\bar{I}_0} + \frac{I_j}{\bar{I}_j} - \frac{S \bar{I}_0 I_j}{\bar{S} I_0 \bar{I}_j}= &  \left( 1 - \frac{\bar{S}}{S} + \log \frac{\bar{S}}{S} \right) + \left( 1 - \frac{S \bar{I}_0 I_j}{\bar{S} I_0 \bar{I}_j} + \log \frac{S I_j \bar{I}_0}{\bar{S} \bar{I}_j I_0} \right) \\ &  \qquad + \left( \frac{I_j}{\bar{I}_j} - \log \frac{I_j}{\bar{I}_j} - \frac{I_0}{\bar{I}_0} + \log \frac{I_0}{\bar{I}_0} \right) , \end{aligned} \end{aligned}$$

and, again, the first two terms under parentheses are non-positive.

Thus, as $\tau _0, \tau _1, \tau _2$ are positive constants, we get

A18 $$\begin{aligned} \begin{aligned} \dot{V}&\le \tau _0 \sum \limits _{j=1}^2 k_j \bar{S} \bar{I}_j \left( \frac{I_j}{\bar{I}_j} - \log \frac{I_j}{\bar{I}_j} - \frac{I_0}{\bar{I}_0} + \log \frac{I_0}{\bar{I}_0} \right) \\&\qquad + \tau _1 (1-p) \bar{I}_0 \left( \frac{I_0}{\bar{I}_0} - \log \frac{I_0}{\bar{I}_0} - \frac{I_1}{\bar{I}_1} + \log \frac{I_1}{\bar{I}_1} \right) \\&\qquad + \tau _2 p \bar{I}_0 \left( \frac{I_0}{\bar{I}_0} - \log \frac{I_0}{\bar{I}_0} - \frac{I_2}{\bar{I}_2} + \log \frac{I_2}{\bar{I}_2} \right) \\ \\&= - \left[ \tau _0 \bar{S} \left( k_1 \bar{I}_1 + k_2 \bar{I}_2 \right) - \tau _1 (1-p) \bar{I}_0 - \tau _2 p \bar{I}_0 \right] \phi _0 \\&\qquad + \left[ \tau _0 k_1 \bar{S} \bar{I}_1 - \tau _1 (1-p) \bar{I}_0 \right] \phi _1 \\&\qquad + \left[ \tau _0 k_2 \bar{S} \bar{I}_2 - \tau _2 p \bar{I}_0 \right] \phi _2, \end{aligned} \end{aligned}$$

where

A19 $$\begin{aligned} \phi _k := \frac{I_k}{\bar{I}_k} - \log \frac{I_k}{\bar{I}_k}, \quad k \in \{0,1,2\}. \end{aligned}$$

We therefore see that if $\tau _0, \tau _1, \tau _2$ are such that the coefficients associated to functions $\phi _0, \phi _1, \phi _2$ in Eq. (A18) cancel out, then we will have $\dot{V} \le 0$. The conditions for this to happen are

A20 $$\begin{aligned} \begin{aligned} 0&= \tau _0 k_1 \bar{S} \bar{I}_1 - \tau _1 (1-p) \bar{I}_0 \\ 0&= \tau _0 k_2 \bar{S} \bar{I}_2 - \tau _2 p \bar{I}_0, \end{aligned} \end{aligned}$$

so we can take

A21 $$\begin{aligned} \tau _0 = 1, \quad \tau _1 = \frac{k_1 \bar{S} \bar{I}_1}{(1-p) \bar{I}_0}, \quad \tau _2 = \frac{k_2 \bar{S} \bar{I}_2}{p \bar{I}_0}. \end{aligned}$$

Using the fixed-point equations (A10), (A13) these constants are

A22 $$\begin{aligned} \tau _0 = 1, \quad \tau _1 = \frac{k_1}{(m+r) K}, \quad \tau _2 = \frac{k_2}{(m+m_d) K}, \end{aligned}$$

which are clearly positive. We conclude that $\dot{V} \le 0$.

It remains to be shown that the only invariant set in $\mathring{\Omega } \cap \{\dot{V}= 0\}$ is $\{\bar{P}\}$. From the above derivation we see that $\dot{V}=0$ implies that the non-positive terms that were removed to obtain bound (A18) have to be exactly 0, that is,

A23 $$\begin{aligned} S = \bar{S} \qquad \text {and} \qquad I_j = \frac{\bar{I}_j}{\bar{I}_0} I_0 \; \text { for } j \in \{1,2\}. \end{aligned}$$

Notice that at any point belonging to an invariant set contained in $\{S= \bar{S}\}$, the temporal derivative of *S* has to be 0. Thus, in $\mathring{\Omega } \cap \{\dot{V}= 0\}$,

A24 $$\begin{aligned} 0 = \dot{S} = b - m \bar{S} - I_0 = \bar{I}_0 - I_0, \end{aligned}$$

so $$I_0 = \bar{I}_0$$ and

A25 $$\begin{aligned} I_j = \frac{\bar{I}_j}{\bar{I}_0} I_0 = \bar{I}_j \quad \text {for } j \in \{1,2\}. \end{aligned}$$

The only point fulfilling the above conditions is $\bar{P}$. This conlcudes the proof. □

### Proposition 3

Consider *n* strains with phenotypes $x_1, \dots , x_n$ and the (2n+1)-dimensional system

A26 $$\begin{aligned} \begin{aligned} S'(t)&= b - m S (t) - \left( \sum \limits _{i=1}^n k_1(x_i) I_1 (t;x_i)+k_2(x_i) I_2 (t;x_i) \right) S (t) \\ I_1'(t;x_i)&= (1-p) \left( k_1(x_i) I_1 (t;x_i)+k_2 I_2 (t;x_i) \right) S(t) - m I_1(t;x_i)-r I_1(t;x_i)\\ I_2'(t;x_i)&= p \left( k_1(x_i) I_1 (t;x_i)+k_2(x_i) I_2 (t;x_i) \right) S(t) -m I_2(t;x_i) - m_d(x_i) I_2(t;x_i) \\ \end{aligned} . \end{aligned}$$

where $i \in \{1,\cdots ,n\}$ in the last two equations. For every phenotype *x* we define

A27 $$\begin{aligned} K(x):=(1-p)\frac{k_1(x)}{m+r}+p\frac{k_2(x)}{m+m_d(x)}. \end{aligned}$$

If $b K(x_1) > m$ and $K(x_1) > K(x_i)$ for all i≠1, then the point

A28 $$\begin{aligned} \bar{P} = (\bar{S}(x_1), \, \bar{I}_1(x_1), \, \bar{I}_2(x_1), \, 0, \, \cdots , \, 0), \end{aligned}$$

with

A29 $$\begin{aligned} \begin{aligned} \bar{S}(x_1)&= 1/K(x_1),\\ \bar{I}_1(x_1)&= \frac{1-p}{m+r} \left( b - \frac{m}{K(x_1)} \right) ,\\ \bar{I}_2(x_1)&= \frac{p}{m+m_d(x_1)} \left( b - \frac{m}{K(x_1)} \right) , \end{aligned} \end{aligned}$$

is an endemic equilibrium of system (A26) and it is globally asymptotically stable in the region

$$ \begin{aligned} \Omega&= \{ (S, I_1(x_1), I_2(x_1), \cdots , I_1(x_n), I_2(x_n) \in \mathbb {R}^{2n+1} \\&\qquad \qquad \,:\, S, I_1(x_1), I_2(x_1) > 0, I_1(x_i), I_2(x_i) \ge 0 \text { for } i \ge 2 \}. \end{aligned} $$

### Proof

It is straightforward to see that $\bar{P}$ is an equilibrium of (A26). Moreover,

$$ \bar{I}_1(x_1), \bar{I}_2(x_1) > 0$$

(the equilibrium is endemic) and $\bar{P} \in \Omega$ because $b K(x_1) > m$.

To prove that $\bar{P}$ is globally asymptotically stable in Ω we will proceed in a similar way as we did in Proposition 2. Consider the Lyapunov function

A30 $$\begin{aligned} \begin{aligned} V(S,&I_1(x_1), I_2(x_1), \cdots , I_1(x_n),I_2(x_n)) \\&\qquad \qquad = \tau _0( S - \bar{S}(x_1) \log S ) \\&\qquad \qquad + \tau _1( I_1(x_1) - \bar{I_1}(x_1) \log I_1(x_1) ) + \tau _2( I_2(x_1) - \bar{I_2}(x_1) \log I_2(x_1) ) \\&\qquad \qquad + c_1^2 I_1(x_2) + c_2^2 I_2(x_2) + \cdots + c_1^n I_1(x_n) + c_2^n I_2(x_n), \end{aligned} \end{aligned}$$

where $\tau _0, \tau _1, \tau _2, c_1^2, c_2^2, \cdots , c_1^n, c_2^n$ are positive constants to be determined.

Clearly, in Ω function *V* has a global minimum at $\bar{P}$. We have to prove that, in Ω,

$$ \dot{V} = \frac{\text {d}}{\text {d}t} V \left( S(t),I_1(t;x_1),I_2(t;x_1), \cdots , I_1(t;x_n),I_2(t;x_n) \right) \le 0 \quad \forall t $$

and that the only invariant set within the set $\{ \dot{V} = 0\}$ is the singleton $\{\bar{P}\}$. Then we will have, by LaSalle’s invariance principle, that $\{\bar{P}\}$ is globally asymptotically stable in Ω.

Let us introduce the auxiliary variables

A31 $$\begin{aligned} I_0(t;x_i) := \left( k_1(x_i) I_1 (t;x_i) + k_2(x_i) I_2 (t;x_i) \right) S (t), \end{aligned}$$

and let $\bar{I}_0(x_i)$ refer to the associated equilibria:

A32 $$\begin{aligned} \bar{I}_0(x_1) \,=\, \left( k_1(x_1) \bar{I}_1(x_1) + k_2 \bar{I}_2(x_1) \right) \bar{S}(x_1) \,=\, b-m \bar{S}(x_1), \qquad \bar{I}_0(x_i) = 0 \text { for } i \ge 2. \end{aligned}$$

Differenciating $V = V( S(t), I_1(t;x_1),I_2(t;x_1),\cdots , I_1(t;x_n),I_2(t;x_n))$ with respect to time we have

A33 $$\begin{aligned} \begin{array}{lll} \dot{V} & =& \displaystyle \frac{\partial V}{\partial S} \dot{S} +\frac{\partial V}{\partial I_1} \dot{I}_1(x_1) +\frac{\partial V}{\partial I_2} \dot{I}_2(x_1) +\cdots +\frac{\partial V}{\partial I_1} \dot{I}_1(x_n) +\frac{\partial V}{\partial I_2} \dot{I}_2(x_n) \\ \\ & =& \displaystyle \tau _0 \left( 1 - \frac{\bar{S}(x_1)}{S} \right) \left( b - m S - \sum \limits _{i=1}^n I_0(x_i) \right) \\ & & \displaystyle + \tau _1 \left( 1 - \frac{\bar{I}_1(x_1)}{I_1(x_1)} \right) \left( (1-p) I_0(x_1) - (m+r) I_1(x_1) \right) \\ & & \displaystyle + \tau _2 \left( 1 - \frac{\bar{I}_2(x_1)}{I_2(x_1)} \right) \left( p I_0(x_1) - (m+m_d(x_1)) I_2(x_1) \right) \\ & & + \displaystyle \sum \limits _{i=2}^n c_1^i \left( (1-p) I_0(x_i) - (m+r) I_1(x_i) \right) \\ & & + \displaystyle \sum \limits _{i=2}^n c_2^i \left( p I_0(x_i) - (m+m_d(x_i)) I_2(x_i) \right) \\ \\ & =& \displaystyle \tau _0 T_0 + \tau _1 \, T_1 + \tau _2 \, T_2 +\displaystyle \sum \limits _{i=2}^n c_1^i \left( (1-p) I_0(x_i) - (m+r) I_1(x_i) \right) \\ & & + \displaystyle \sum \limits _{i=2}^n c_2^i \left( p I_0(x_i) - (m+m_d(x_i)) I_2(x_i) \right) . \end{array} \end{aligned}$$

We start analyzing the terms $T_0, T_1, T_2$. Using the fixed point equations (A29), (A28), (A32) we can express

A34 $$\begin{aligned} \begin{array}{lll} T_0 \\ & =& \displaystyle - \frac{m}{S} (S-\bar{S}(x_1))^2 \\ & & + \sum \limits _{j=1}^2 k_j(x_1) \bar{S}(x_1) \bar{I}_j(x_1) \left( 2 - \frac{\bar{S}(x_1)}{S} - \frac{I_0(x_1)}{\bar{I}_0(x_1)} + \frac{I_j(x_1)}{\bar{I}_j(x_1)} - \frac{S \bar{I}_0(x_1) I_j(x_1)}{\bar{S}(x_1) I_0(x_1) \bar{I}_j(x_1)} \right) \\  & & \displaystyle + \left( \frac{\bar{S}(x_1)}{S} - 1 \right) \sum \limits _{i=2}^n I_0(x_i) \\ T_1 & =& \displaystyle (1-p) \bar{I}_0(x_1) \left( 1 + \frac{I_0(x_1)}{\bar{I}_0(x_1)} - \frac{I_1(x_1)}{\bar{I}_1(x_1)} - \frac{I_0(x_1) \bar{I}_1(x_1)}{\bar{I}_0(x_1) I_1(x_1)} \right) \\ T_2 & =& \displaystyle p \bar{I}_0(x_1) \left( 1 + \frac{I_0(x_1)}{\bar{I}_0(x_1)} - \frac{I_2(x_1)}{\bar{I}_2(x_1)} - \frac{I_0(x_1) \bar{I}_2(x_1)}{\bar{I}_0(x_1) I_2(x_1)} \right) . \end{array} \end{aligned}$$

Denoting, for simplicity, $\bar{S}:=\bar{S}(x_1)$, $I_0:=I_0(x_1)$, $\bar{I}_0:= \bar{I}_0(x_1)$, $I_j:=I_j(x_1)$, $\bar{I}_j:=\bar{I}_j(x_1)$, and using that all of them are positive in Ω, we can rewrite, for $j \in \{1,2\}$,

A35 $$\begin{aligned} 1 + \frac{I_0}{\bar{I}_0} - \frac{I_j}{\bar{I}_j} - \frac{I_0 \bar{I}_j}{\bar{I}_0 I_j} = \left( 1 - \frac{I_0 \bar{I}_j}{\bar{I}_0 I_j} + \log \frac{I_0 \bar{I}_j}{\bar{I}_0 I_j}\right) + \left( \frac{I_0}{\bar{I}_0} - \log \frac{I_0}{\bar{I}_0} - \frac{I_j}{\bar{I}_j} + \log \frac{I_j}{\bar{I}_j} \right) . \end{aligned}$$

The term under the first parenthesis is non-positive because $1-x+\log x \le 0$ for x>0.

Similarly,

A36 $$\begin{aligned} \begin{aligned} 2 - \frac{\bar{S}}{S} - \frac{I_0}{\bar{I}_0} + \frac{I_j}{\bar{I}_j} - \frac{S \bar{I}_0 I_j}{\bar{S} I_0 \bar{I}_j}&= \left( 1 - \frac{\bar{S}}{S} + \log \frac{\bar{S}}{S} \right) + \left( 1 - \frac{S \bar{I}_0 I_j}{\bar{S} I_0 \bar{I}_j} + \log \frac{S \bar{I}_0 I_j}{\bar{S} I_0 \bar{I}_j} \right) \\&\qquad + \left( \frac{I_j}{\bar{I}_j} - \log \frac{I_j}{\bar{I}_j} - \frac{I_0}{\bar{I}_0} + \log \frac{I_0}{\bar{I}_0} \right) , \end{aligned} \end{aligned}$$

and, again, the first two terms under parentheses are non-positive.

Also, $K(x_1) > K(x_i)$ for all i≥2 by hypothesis, and this implies $\bar{S}(x_1) = 1/K(x_1) < 1/K(x_i)$ for all i≥2.

Thus, we can bound the terms $T_0$, $T_1$, $T_2$ by

A37 $$\begin{aligned} \begin{array}{lll} T_0 & \le & \displaystyle \sum \limits _{j=1}^2 k_j(x_1) \bar{S}(x_1) \bar{I}_j(x_1) \left( \frac{I_j(x_1)}{\bar{I}_j(x_1)} - \log \frac{I_j(x_1)}{\bar{I}_j(x_1)} - \frac{I_0(x_1)}{\bar{I}_0(x_1)} + \log \frac{I_0(x_1)}{\bar{I}_0(x_1)} \right) \\ & & \qquad \displaystyle + \sum \limits _{i=2}^n \frac{I_0(x_i)}{K(x_i)S} \\ T_1 & \le & \displaystyle (1-p) \bar{I}_0(x_1) \left( \frac{I_0(x_1)}{\bar{I}_0(x_1)} - \log \frac{I_0(x_1)}{\bar{I}_0(x_1)} - \frac{I_1(x_1)}{\bar{I}_1(x_1)} + \log \frac{I_1(x_1)}{\bar{I}_1(x_1)} \right) \\ T_2 & \le & \displaystyle p \bar{I}_0(x_1) \left( \frac{I_0(x_1)}{\bar{I}_0(x_1)} - \log \frac{I_0(x_1)}{\bar{I}_0(x_1)} - \frac{I_2(x_1)}{\bar{I}_2(x_1)} + \log \frac{I_2(x_1)}{\bar{I}_2(x_1)} \right) \end{array} \end{aligned}$$

and, since $\tau _0, \tau _1, \tau _2$ are positive constants, we get

A38 $$\begin{aligned} \dot{V}&\le \tau _0 \left[ \sum \limits _{j=1}^2 k_j(x_1) \bar{S}(x_1) \bar{I}_j(x_1) \left( \frac{I_j(x_1)}{\bar{I}_j(x_1)} - \log \frac{I_j(x_1)}{\bar{I}_j(x_1)} - \frac{I_0(x_1)}{\bar{I}_0(x_1)} + \log \frac{I_0(x_1)}{\bar{I}_0(x_1)} \right) + \sum \limits _{i=2}^n \frac{I_0(x_i)}{K(x_i) S} \right] \nonumber \\&\quad + \tau _1 \, (1-p) \bar{I}_0(x_1) \left( \frac{I_0(x_1)}{\bar{I}_0(x_1)} - \log \frac{I_0(x_1)}{\bar{I}_0(x_1)} - \frac{I_1(x_1)}{\bar{I}_1(x_1)} + \log \frac{I_1(x_1)}{\bar{I}_1(x_1)} \right) \nonumber \\&\quad +\tau _2 \, p \bar{I}_0(x_1) \left( \frac{I_0(x_1)}{\bar{I}_0(x_1)} - \log \frac{I_0(x_1)}{\bar{I}_0(x_1)} - \frac{I_2(x_1)}{\bar{I}_2(x_1)} + \log \frac{I_2(x_1)}{\bar{I}_2(x_1)} \right) \nonumber \\&\quad + \displaystyle \sum \limits _{i=2}^n c_1^i \left( (1-p) I_0(x_i) - (m+r) I_1(x_i) \right) + \sum \limits _{i=2}^n c_2^i \left( p I_0(x_i) - (m+m_d(x_i)) I_2(x_i) \right) \nonumber \\&= - \left[ \tau _0 \, \bar{S}(x_1) \left( k_1(x_1) \bar{I}_1(x_1) + k_2(x_1) \bar{I}_2(x_1) \right) - \tau _1 (1-p) \bar{I}_0(x_1) - \tau _2 \, p \bar{I}_0(x_1) \right] \phi _0 \nonumber \\&\quad + \left[ \tau _0 \, k_1(x_1) \bar{S}(x_1) \bar{I}_1(x_1) - \tau _1 (1-p) \bar{I}_0(x_1) \right] \phi _1 \nonumber \\&\quad + \left[ \tau _0 \, k_2(x_1) \bar{S}(x_1) \bar{I}_2(x_1) - \tau _2 \, p \bar{I}_0(x_1) \right] \phi _2 \nonumber \\&\quad + \sum \limits _{i=2}^n \left[ c_1^i (1-p) + c_2^i p - \tau _0 \right] I_0(x_i) \nonumber \\&\quad + \sum \limits _{i=2}^n \left[ \frac{\tau _0}{K(x_i)} - \frac{ c_1^i (m+r) I_1(x_i) + c_2^i (m+m_d(x_i)) I_2(x_i)}{ \frac{I_0(x_i)}{S}} \right] \frac{I_0(x_i)}{S}, \end{aligned}$$

where

A39 $$\begin{aligned} \phi _k := \frac{I_k(x_1)}{\bar{I}_k(x_1)} - \log \frac{I_k(x_1)}{\bar{I}_k(x_1)}, \quad k \in \{0,1,2\}. \end{aligned}$$

We therefore see that if the constants $\tau _0, \tau _1, \tau _2, c_1^i, c_2^i$ are positive and such that all the expressions inside the brackets in Eq. (A38) cancel out, then we will have $\dot{V} \le 0$. The conditions for this to happen are

A40 $$\begin{aligned} \begin{aligned} 0&= \tau _0 k_1(x_1) \bar{S}(x_1) \bar{I}_1(x_1) - \tau _1 (1-p) \bar{I}_0(x_1) \\ 0&= \tau _0 k_2(x_1) \bar{S}(x_1) \bar{I}_2(x_1) - \tau _2 p \bar{I}_0(x_1) \\ 0&= c_1^i (1-p) + c_2^i p - \tau _0 \\ 0&= \frac{\tau _0}{K(x_i)} - \frac{ c_1^i (m+r) I_1(x_i) + c_2^i (m+m_d(x_i)) I_2(x_i)}{ k_1(x_i) I_1(x_i) + k_2(x_i) I_2(x_i)}. \end{aligned} \end{aligned}$$

Without loss of generality we can take

$$\begin{aligned} \tau _0 = 1. \end{aligned}$$

Then, from the first two equations we find

A41 $$\begin{aligned} \tau _1 = \frac{k_1(x_1) \bar{S}(x_1) \bar{I}_1(x_1)}{(1-p) \bar{I}_0(x_1)}, \quad \tau _2 = \frac{k_2(x_1) \bar{S}(x_1) \bar{I}_2(x_1)}{p \bar{I}_0(x_1)}. \end{aligned}$$

Using the fixed-point equations (A29), (A28), (A32) these constants are

A42 $$\begin{aligned} \tau _1 = \frac{k_1(x_1)}{(m+r) K(x_1)}, \quad \tau _2 = \frac{k_2(x_1)}{(m+m_d(x_1)) K(x_1)}, \end{aligned}$$

which are clearly positive. The last equation imposes

A43 $$\begin{aligned} 1 = \frac{ c_1^i (m+r) K(x_i) I_1(x_i) + c_2^i (m+m_d(x_i)) K(x_i) I_2(x_i)}{ k_1(x_i) I_1(x_i) + k_2(x_i) I_2(x_i)}, \end{aligned}$$

so we can set

A44 $$\begin{aligned} c_1^i = \frac{k_1(x_i)}{(m+r) K(x_i)}, \quad c_2^i = \frac{k_2(x_i)}{(m+m_d(x_i)) K(x_i)}, \end{aligned}$$

which are also positive. With this setting, the third condition in (A40) is automatically fulfilled and we conclude that $\dot{V} \le 0$ in Ω.

It remains to be shown that the only invariant set in $\{\dot{V} = 0\}$ is $\{\bar{P}\}$. From the above derivation we see that $\dot{V}=0$ implies that the non-positive terms that were removed to obtain bound (A37) have to be exactly 0. Thus,

A45 $$\begin{aligned} S = \bar{S}(x_1), \qquad I_j(x_1) = \frac{\bar{I}_j(x_1)}{\bar{I}_0(x_1)} I_0(x_1) \text { for } j \in {1,2}, \qquad I_0(x_i) = 0 \text { for } i \ge 2. \end{aligned}$$

Notice that at any point belonging to an invariant set contained in $\{S = \bar{S}(x_1)\}$, the temporal derivative of *S* has to be 0. Thus, in $\Omega \cap \{\dot{V} =0\}$,

A46 $$\begin{aligned} 0 = \dot{S} = b - m \bar{S}(x_1) - I_0(x_1) = \bar{I}_0(x_1) - I_0(x_1) \end{aligned}$$

so $$I_0(x_1) = \bar{I}_0(x_1)$$ and

A47 $$\begin{aligned} I_j(x_1) = \frac{\bar{I}_j(x_1)}{\bar{I}_0(x_1)} I_0(x_1) = \bar{I}_j(x_1) \quad \text { for } j \in \{1,2\}. \end{aligned}$$

The only point fulfilling all the above conditions is $\bar{P}$. This concludes the proof. □

### Remark 4

Proposition 3 is not fully surprising in the light of the general result given in Proposition 3.2 of Metz et al. (1996). According to this proposition, and noticing that the environment felt by infected hosts is simply the susceptible population (by environment we mean everything external to an individual affecting its behavior), a mutant population $(I_1(y),I_2(y))$ of type *y* can proliferate within a resident population of type *x* at equilibrium if and only if $\bar{S}(y)<\bar{S}(x)$. This is so because the growth rate of the infected population is increasing with respect to the susceptible population (i.e. the environment). More explicitly: the growth rate of the pair $(I_1,I_2)$ increases with *S* when *S*(*t*) is replaced by a parameter *S* in Eq. (1). Nevertheless, notice that Proposition 3 gives a global result (it says that no matter how large the populations of the different strains are, all but the one with the largest *K* are deemed to extinction), whereas the results in Metz et al. (1996) are local (they focus on whether a mutant strain can grow amidst a large resident population in equilibrium).

### Proposition 4

Let us consider *n* pathogen populations with phenotypes $x_1, \cdots , x_n$ whose dynamics within a host obeys

A48 $$\begin{aligned} \dot{V}_i = \Gamma (V, x_i) V_i \qquad \text {with}\qquad V=\sum _{i=1}^n V_i \quad \text {and} \quad \Gamma (V, x_i) := x_i\beta (V) -\phi (x_i)\mu (V). \end{aligned}$$

Suppose that ϕ(x)>0 for all *x*, $\beta (V), \mu (V) > 0$ for all *V*, $\beta (0) = \mu (0) = 1$, and that Γ(V,x) is strictly decreasing in *V* and negative for *V* large enough, for all *x*. If the ratios $R(x_1), \cdots , R(x_n)$ with $R(x_i)=\frac{x_i}{\phi (x_i)}$ are all different, $R(x_1) > 1$, and $R(x_1) > R(x_i)$ for all i≠1, then the point $\bar{P} = (\bar{V}_1, \cdots , \bar{V}_n)$ with

A49 $$\begin{aligned} \begin{array}{lll} \bar{V}_1 \text { being the only positive solution to } \Gamma ( V_1, x_1 ) = 0, \\ \bar{V}_i = 0 \quad \text {for } i \ge 2, \\ \end{array} \end{aligned}$$

is an equilibrium of system (A48) and it is globally asymptotically stable in the region

$$\begin{aligned} \Omega := \{(V_1, \cdots , V_n) \,:\, V_1 > 0, V_2 \ge 0, \cdots , V_n \ge 0\}. \end{aligned}$$

### Proof

First, we notice that $\Gamma ( V_1, x_1 ) = 0$ has exactly one positive solution. This is so because

$$ \Gamma ( 0, x_1 ) = x_1 \beta (0) - \phi (x_1) \mu (0) = x_1 - \phi (x_1) = \phi (x_1) \left( R(x_1) - 1 \right) > 0, $$

$\Gamma (V,x_1)$ is strictly decreasing in *V* and is negative for *V* large enough. We thus see that $\bar{P}$ exists and it is an equilibrium of (A48).

Now we have to prove that it is globally asymptotically stable in Ω.

We observe that, for i≥2 and for all *V*,

A50 $$\begin{aligned} \begin{aligned} \Gamma (V,x_i)&= x_i \beta (V) - \phi (x_i) \mu (V) \\&= \phi (x_i) \left( R(x_i) \beta (V) - \mu (V) \right) \\&< \phi (x_i) \left( R(x_1) \beta (V) - \mu (V) \right) \\&= \frac{\phi (x_i)}{\phi (x_1)} \left( x_1 \beta (V) - \phi (x_1) \mu (V) \right) \\&= \frac{\phi (x_i)}{\phi (x_1)} \Gamma (V,x_1). \end{aligned} \end{aligned}$$

Suppose that, at a given time, $V > \bar{V}_1$. Then, using Eq. (A50) and the fact that $\Gamma (V,x_i)$ is strictly decreasing in *V*, we have

A51 $$\begin{aligned} \dot{V} = \Gamma (V,x_1) V_1 + \sum \limits _{i=2}^n \Gamma (V,x_i) V_i < \Gamma (\bar{V}_1,x_1) V_1 + \sum \limits _{i=2}^n \frac{\phi (x_i)}{\phi (x_1)} \Gamma (\bar{V}_1,x_1) V_i = 0, \end{aligned}$$

so *V* either enters or approaches asymptotically the positively invariant region

$$ \tilde{\Omega } = \Omega \cap \left\{ (V_1, \cdots , V_n) \,:\, V \le \bar{V}_1\right\} . $$

Let us define $\tilde{\Omega }_\varepsilon \subset \tilde{\Omega }$ as the bounded and closed set

$$ \tilde{\Omega }_\varepsilon = \Omega _\varepsilon \cap \left\{ (V_1, \cdots , V_n) \,:\, V \le \bar{V}_1\right\} , $$

where

$$ \Omega _\varepsilon := \{(V_1, \cdots , V_n) \,:\, V_1 \ge \varepsilon , V_2 \ge 0, \cdots , V_n \ge 0\}.$$

We will see that, if $0< \varepsilon < \bar{V}_1$, all trajectories starting within $\tilde{\Omega }_\varepsilon$ converge to $\bar{P}$. Then, since every point in $\tilde{\Omega }$ is contained in a set $\tilde{\Omega }_\varepsilon$ for some ε, the same will be true for all trajectories starting within $\tilde{\Omega }$.

Let ε be such that $0<\varepsilon < \bar{V}_1$. Within $\tilde{\Omega }_\varepsilon$, we define the Lyapunov function

$$ L(V_1, \cdots , V_n) = \frac{1}{2} (V_1 - \bar{V}_1)^2. $$

*L* is minimal when $V_1 = \bar{V}_1$, and in $\tilde{\Omega }_\varepsilon$ this is only possible at $(V_1, V_2, \cdots , V_n) = (\bar{V}_1, 0, \cdots , 0) = \bar{P}$. Also, in $\tilde{\Omega }_\varepsilon$ the temporal derivative of $L(V_1(t), \cdots , V_n(t))$ is

A52 $$\begin{aligned} \dot{L} = (V_1 - \bar{V}_1) \dot{V}_1 \le 0 \end{aligned}$$

because $$0 < V_1 \le \bar{V}_1$$ and

A53 $$\begin{aligned} \dot{V}_1 = \Gamma (V, x_1 ) V_1 \ge \Gamma (\bar{V}_1, x_1 ) V_1 = 0. \end{aligned}$$

Finally, $\dot{L}=0$ in $\tilde{\Omega }_\varepsilon$ implies $V_1 = \bar{V}_1$ or $\dot{V}_1 = 0$. In the first case, we have

A54 $$\begin{aligned} \bar{V}_1 \ge V = \sum \limits _{i=1}^n V_i \ge V_1 = \bar{V}_1, \end{aligned}$$

so necessarily $V=\bar{V_1}$. In the second case,

A55 $$\begin{aligned} 0 = \dot{V}_1 = \Gamma (V,x_1) V_1, \end{aligned}$$

so $\Gamma (V,x_1) = 0$ and necessarily $V= \bar{V}_1$. This implies

A56 $$\begin{aligned} \tilde{\Omega }_\varepsilon \cap \{ \dot{L}=0\} \;=\; \tilde{\Omega }_\varepsilon \cap \{V = \bar{V}_1\}. \end{aligned}$$

We now show that within any invariant set contained in $\tilde{\Omega }_\varepsilon \cap \{V = \bar{V}_1 \}$, necessarily $V_i = 0$ for i≥2. If this were not true, there would exist an index *k* such that $V_k > 0$. This, together with the invariance property in the set (and using Eq. (A50)), would imply

A57 $$\begin{aligned} \begin{array}{llllllllll} 0= &  \dot{V}= &  \displaystyle \sum \limits _{i=1}^n \Gamma (\bar{V}_1,x_i) V_i< &  \displaystyle \Gamma (\bar{V}_1,x_1) V_1 + \sum \limits _{i=2}^n \frac{\phi (x_i)}{\phi (x_1)} \Gamma (\bar{V}_1,x_1) V_i= &  0, \end{array} \end{aligned}$$

which is absurd.

We conclude that in any invariant set within $\tilde{\Omega }_\varepsilon \cap \{ \dot{L}=0\}$, we have $V = \bar{V}_1$ and $V_i = 0$ for i≥2. The only set with these properties is $\{\bar{P}\}$. This concludes the proof. □
