# Correction: Virulence Evolution in Host Populations with Heterogeneous Immune Responses: The Weakening of the Uncommon Weak

**DOI:** 10.1007/s11538-026-01778-1

**Published:** 2026-10-20

**Authors:** Carles Barril, Marina Vegué

**Affiliations:** 1 Departament de Matemàtiques, Universitat Autònoma de Barcelona, Barcelona, Spain; 2 Departament de Matemàtiques, Universitat Politècnica de Catalunya, Barcelona, Spain

**Correction: Bulletin of Mathematical Biology (2026) 88:109** **https://doi.org/10.1007/s11538-026-01673-9**

In the original article the Acknowledgements section is incomplete. The Acknowledgements section should read:

"M.V. acknowledges financial support through the grant María Zambrano - UPC from the Spanish Ministry of Universities and the European Union - NextGenerationEU. This work was partially supported by the research projects PID-2021-123733NB-I00 and PID-2021-122954NB-100, and by Grant PID2024-155942NB-I00 funded by MICIU/AEI/10.13039/501100011033 and ERDF, UE."

